# Metabolic programming determines the lineage-differentiation fate of murine bone marrow stromal progenitor cells

**DOI:** 10.1038/s41413-019-0076-5

**Published:** 2019-11-14

**Authors:** Michaela Tencerova, Elizabeth Rendina-Ruedy, Ditte Neess, Nils Færgeman, Florence Figeac, Dalia Ali, Morten Danielsen, Anders Haakonsson, Clifford J. Rosen, Moustapha Kassem

**Affiliations:** 1 Department of Molecular Endocrinology, University of Southern Denmark and Odense University Hospital, 5000 Odense, Denmark; 2 Center for Molecular Medicine, Maine Medical Center Research Institute, Scarborough, ME 04074 USA; 3 Villum Center for Analytical Biosciences, Department of Biochemistry and Molecular Biology, University of Southern Denmark, 5230 Odense, Denmark; 4 MSOmics, 2950 Copenhagen, Denmark; 5 Department of Cellular and Molecular Medicine, Novo Nordisk Foundation Center for Stem Cell Biology (DanStem), University of Copenhagen, 2200 Copenhagen, Denmark

**Keywords:** Bone, Fat metabolism

## Abstract

Enhanced bone marrow adipogenesis and impaired osteoblastogenesis have been observed in obesity, suggesting that the metabolic microenvironment regulates bone marrow adipocyte and osteoblast progenitor differentiation fate. To determine the molecular mechanisms, we studied two immortalized murine cell lines of adipocyte or osteoblast progenitors (BMSCs^adipo^ and BMSCs^osteo^, respectively) under basal and adipogenic culture conditions. At baseline, BMSCs^adipo^, and BMSCs^osteo^ exhibit a distinct metabolic program evidenced by the presence of specific global gene expression, cellular bioenergetics, and metabolomic signatures that are dependent on insulin signaling and glycolysis in BMSCs^osteo^ versus oxidative phosphorylation in BMSCs^adipo^. To test the flexibility of the metabolic program, we treated BMSCs^adipo^ with parathyroid hormone, S961 (an inhibitor of insulin signaling) and oligomycin (an inhibitor of oxidative phosphorylation). The treatment induced significant changes in cellular bioenergetics that were associated with decreased adipocytic differentiation. Similarly, 12 weeks of a high-fat diet in mice led to the expansion of adipocyte progenitors, enhanced adipocyte differentiation and insulin signaling in cultured BMSCs. Our data demonstrate that BMSC progenitors possess a distinct metabolic program and are poised to respond to exogenous metabolic cues that regulate their differentiation fate.

## Introduction

Bone marrow (BM) is a heterogeneous organ that contains, in addition to hematopoietic stem cells, BM stromal (also known as skeletal or mesenchymal) stem cells (BMSCs) and their descendent progenitors of adipocyte (AD) and osteoblast (OB) lineages that give rise to BM adipose tissue (BMAT) and bone, respectively.^1–5^

The differentiation of BMSCs is regulated by a variety of extracellular factors present in the BM microenvironment.^3,6^ Metabolic changes in obesity, type 2 diabetes, and anorexia nervosa lead to differential effects on BMSC differentiation capacity and are associated with increased BMAT formation,^7^ suggesting that the metabolic state of the organism regulates BMSC differentiation fate.

In a number of stem cell models, such as pluripotent embryonic stem cells, hematopoietic, muscle stem cells^8–10^ and immune cells,^11^ cellular differentiation and functions are determined by the cellular metabolic and bioenergetic state.^12^ Pluripotent embryonic stem cells prefer anabolic glycolysis, which is also the preferred metabolic process of rapidly proliferating cells.^8,13^ In addition, hematopoietic progenitor cells exhibit differentiation-dependent use of glycolysis or oxidative phosphorylation (OxPhos).^14,15^ Similarly, during extramedullary AD differentiation, a switch in the bioenergetic program from glycolysis to OxPhos has been observed and provides AD progenitors with sufficient energy for histone acetylation and activation of the lipogenic and adipogenic program.^16^ On the other hand, during mid to late OB differentiation, glycolysis is the preferred bioenergetic mechanism.^17^ These data suggest that the differentiation responses of progenitor cells to metabolic changes in their microenvironment are dependent on their bioenergetic state and substrate choice.^18^ However, it is not known whether a specific metabolic program of BMSC progenitors exists and whether it determines their differentiation responses.

To address this question, we employed two immortalized murine cell lines of progenitor cells that are committed towards either AD or OB (BMSCs^adipo^ and BMSCs^osteo^, respectively) as they represent two alternative differentiation choices of BMSCs in vivo. These cell lines have previously been established and characterized in our laboratory.^19–21^ We tested the hypothesis that there exists a distinct metabolic program in BMSC progenitors that determines their differentiation responses to exogenous metabolic cues. Applying the global approaches of transcriptional and metabolomics analyses, we demonstrate that AD versus OB progenitors exhibit a unique bioenergetic profile and intrinsic metabolic program that is responsive to the exogenous cue of insulin and regulates their differentiation outcome. We corroborated these findings by reporting similar changes in BMSC differentiation capacity in vivo under conditions of metabolic changes related to high-fat diet (HFD)-induced obesity in mice.

## Results

### BMSCs^adipo^ and BMSCs^osteo^ progenitors exhibit a unique gene expression profile

**Fig. 1:**
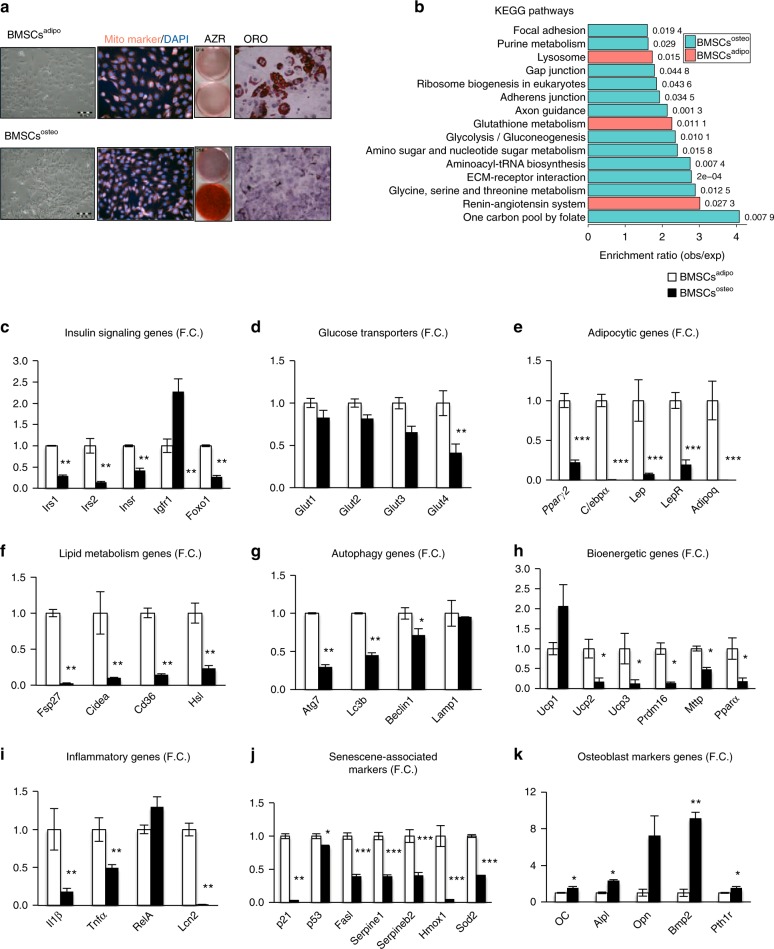
BMSCs^adipo^ and BMSCs^osteo^ progenitors exhibit a unique gene expression profile. **a** Representative pictures of BMSCs^adipo^ and BMSCs^osteo^ with mitochondrial staining using MitoTracker (red), Alizarin staining (AZR) for matrix mineralization and Oil Red O staining (ORO) showing the morphological and differentiation-induced differences between the murine cell lines. **b** Enrichment of genes of functional GO categories from microarray. Gene expression profiling in BMSCs^adipo^ (white bars) and BMSCs^osteo^ cultured in basal conditions (black bars): **c** genes involved in insulin signaling such as *Irs1, Irs2, Insr, Igfr1*, and *Foxo1*; **d** glucose transporters such as *Glut1, Glut2, Glut3*, and *Glut4*; **e** adipocytic genes such as *Pparγ2, C/ebpα, Lep, LepR*, and *Adipoq*; **f** genes involved in lipid metabolism such as *Fsp27, Cidea, Cd36*, and *Hsl*; **g** autophagy genes such as *Atg7, Lc3b, Beclin1*, and *Lamp1*; **h** bioenergetic genes such as *Ucp1, Ucp2, Ucp3, Prdm16, Mttp*, and *Pparα*; **i** inflammatory genes such as *Il1β, Tnfα, Mcp1, RelA*, and *Lcn2*; **j** senescence-associated markers such as *p21, p53; Fasl, Serpine1, Serpineb2, Hmox1*, and *Sod2*; **k** osteoblast marker genes such as *Oc, Opn, Alpl*, *Bmp2*, and *Pthr1*. Data are presented as the mean fold change (F.C.) of gene expression normalized to BMSCs^adipo^ expression ± SEM, (*n* = 3 per group); (*P < 0.05, **P < 0.01; ***P < 0.001: BMSCs^adipo^ vs BMSCs^osteo^, two-tailed unpaired Student’s *t* test)

BMSC cultures represent a heterogeneous population of cells that include stem cells and their descendent progenitors at different stages of differentiation.^4,22^ To avoid confounding variables mediated by different cell populations present within primary cultures, we employed two immortalized murine lines that were functionally defined by their ability to differentiate into either AD(BMSCs^adipo^) or OB (BMSCs^osteo^)^19^ (Fig. 1a). To identify the presence of a molecular signature of bioenergetic and metabolic programs, we performed global gene expression profiling under basal conditions^20,21^ that revealed differential enrichment of genes associated with the following metabolic pathways: insulin signaling, PPAR signaling, fatty acid oxidation, glycolysis and cell adhesion, cell cycle, immune system, and purine metabolism in BMSCs^adipo^ versus BMSCs^osteo^ (Fig. 1b and Fig. S1). Concordant changes in the expression of a representative group of genes were confirmed by qRT-PCR (Fig. 1c–k). Compared with BMSCs^osteo^, gene expression profiling of BMSCs^adipo^ revealed increased expression of insulin-responsive genes (*Irs1, Irs2, Insr*, and *Foxo1*) (Fig. 1c), glucose transporters (*Glut4*) (Fig. 1d), adipocytic genes (*Pparγ2, C/epbα, Lep*, and *Adipoq*) (Fig. 1e), lipid metabolism genes (*Fsp27, Cidea, Cd36*, and *Hsl*) (Fig. 1f), autophagy (*Atg7, Lc3b*, and *Beclin1)* (Fig. 1g), bioenergetic genes (*Ucp*, *Prdm16, Mttp*, and *Pparα*) (Fig. 1h), inflammatory genes (*Il1β, Tnfα*, and *Lcn2*) (Fig. 1i), and senescence-associated markers (*p21*, *p53, Fasl, Hmox1*, and *Sod2*) (Fig. 1j). As expected, BMSCs^adipo^ expressed lower levels of osteoblastic genes (Fig. 1k). We employed this molecular signature to test the flexibility of the BMSCs^adipo^ and BMSCs^osteo^ phenotypes in response to adipogenic culture conditions that promote AD differentiation. As shown in Fig. S2a–e, adipogenic culture conditions promoted AD formation only in BMSCs^adipo^ but not in BMSCs^osteo^. In addition, the gene expression profile of adipocytic genes and genes associated with insulin signaling, glucose transporter, lipid metabolism, and autophagy were enhanced in BMSCs^adipo^ but not in BMSCs^osteo^. On the other hand, BMSCs^osteo^ exhibited higher gene expression levels of OB marker genes (*Oc, Alpl, Bmp2*, and *Pth1r*), which were more robust in the presence of OB differentiation induction medium, along with increased Alkaline phosphatase (ALP) activity and no effect on adipocytic marker genes in compared with those in BMSCs^adipo^ (Fig. 1k, Fig. S2f–h). These data corroborate that BMSCs^adipo^ and BMSCs^osteo^ progenitors are committed progenitors poised to respond to metabolic and nutrient stimuli and that they exhibit significant differences in the gene expression levels of metabolic and bioenergetic genes.

### BMSCs^adipo^ and BMSCs^osteo^ progenitors exhibit differential responses to insulin

**Fig. 2:**
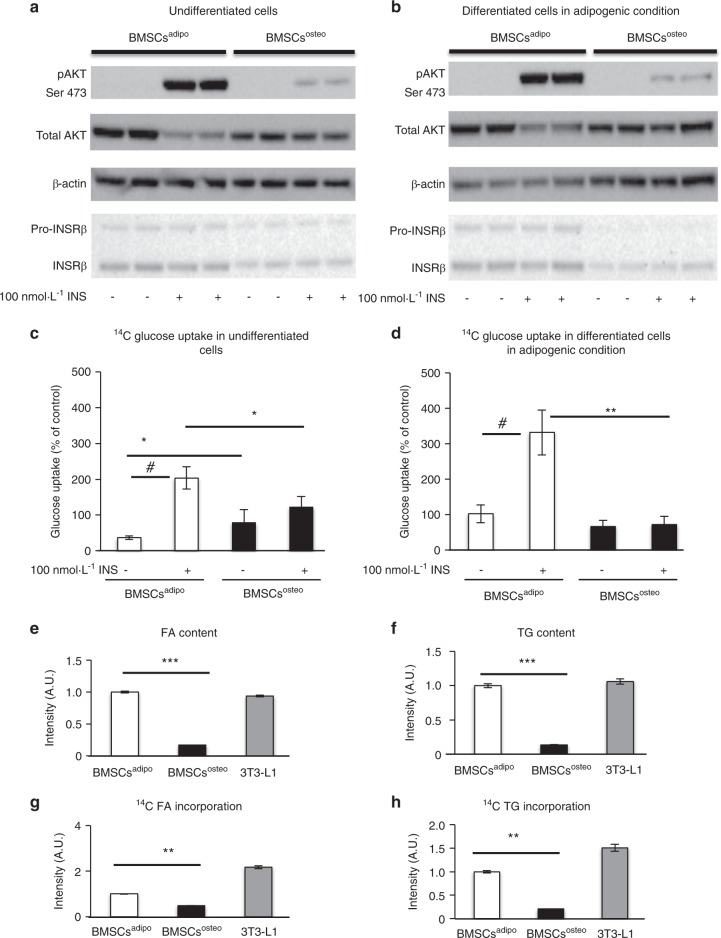
BMSCs^adipo^ and BMSCs^osteo^ progenitors exhibit differential responses to insulin. Representative western blot to evaluate insulin-stimulated (100 nmol·L^–1^, 15 min) phosphorylation of AKT (p-S473AKT) and total AKT, INSRβ **a** in undifferentiated BMSCs^adipo^ and BMSCs^osteo^ and **b** BMSCs^adipo^ and BMSCs^osteo^ differentiated cells in adipogenic conditions (*n* = 3). Insulin-stimulated glucose uptake using a ^14^C glucose tracer after a 24-h incubation **c** in undifferentiated (baseline) BMSCs and **d** BMSCs differentiated under adipogenic conditions from BMSCs^adipo^ and BMSCs^osteo^. Data are presented as the mean of glucose uptake normalized to the baseline of murine 3T3-L1 fibroblasts as a positive control ± SEM from three independent experiments. (*P < 0.05, **P < 0.01: BMSCs^adipo^ vs BMSCs^osteo^, two-tailed unpaired Student’s *t* test; ^#^*P* < 0.05: nonstimulated vs insulin-stimulated cells). Profile of neutral lipids in BMSCs^adipo^, BMSCs^osteo^, and 3T3-L1 cells under insulin stimulation (100 nmol·L^–1^) using thin layer chromatography (TLC): **e** densitometry of triglycerides (TG) and **f** the the fatty acid (FA) content in BMSC differentiated cells under adipogenic conditions. Densitometry of ^14^C FA (**g**) and ^14^C TG (**h**) incorporation in BMSC differentiated cells under adipogenic conditions. Data are presented as the mean of F.C. ± SEM from three independent experiments. (*n* = 3) (*P < 0.05, **P < 0.01: BMSCs^adipo^ vs BMSCs^osteo^, one-way ANOVA)

To investigate the responsiveness of BMSCs^adipo^ and BMSCs^osteo^ to the metabolic environment, we determined the responses to insulin as the major regulator of cellular energy metabolism. BMSCs^adipo^ exhibited higher levels of insulin signaling genes (Fig. 1c) and enhanced responsiveness to insulin compared with those of BMSCs^osteo^ as measured by pAKT(Ser-473)/total AKT (Fig. 2a, b and Fig. S3a, b, left panels), and this occurs at baseline and when cultured under adipogenic conditions. In addition, insulin receptor (INSR) protein levels were higher in BMSCs^adipo^ compared with BMSCs^osteo^ (Fig. 2a, b, Fig. S3a, b, right panels).

### BMSCs^adipo^ exhibit higher insulin-dependent glucose utilization and de novo lipogenesis compared with those of BMSCs^osteo^

Since BMSCs^adipo^ were more insulin-responsive at baseline and under adipogenic conditions, we investigated functional insulin responses in the two immortalized progenitor cell lines (Fig. 2c, d). BMSCs^adipo^ were more responsive to insulin stimulation, as reflected by the higher glucose uptake under baseline and adipogenic culture conditions (Fig. 2c, d). In addition at baseline, glucose uptake (Fig. 2c, d) was higher in BMSCs^osteo^ compared with uptake in BMSCs^adipo^.

At baseline, de novo synthesized lipids determined by thin layer chromatography (TLC) analysis were undetectable (data not shown), while following treatment with adipogenic culture conditions, BMSCs^adipo^ exhibited higher levels of insulin-mediated glucose incorporation into fatty acids (FA) and triglyceride (TG) (Fig. 2e, f), which was similar to what was observed in 3T3-L1 cells.^23^ BMSCs^osteo^ did not accumulate lipids (Fig. 2e, f). Additional studies employing radiolabeled ^14^C glucose detected the incorporation of glucose into lipids (FA and TG), demonstrating de novo lipogenesis in BMSCs^adipo^ and 3T3-L1 but not in BMSCs^osteo^ under adipogenic culture conditions (Fig. 2g, h).

### BMSCs^adipo^ and BMSCs^osteo^ exhibit a distinct bioenergetic profile

**Fig. 3:**
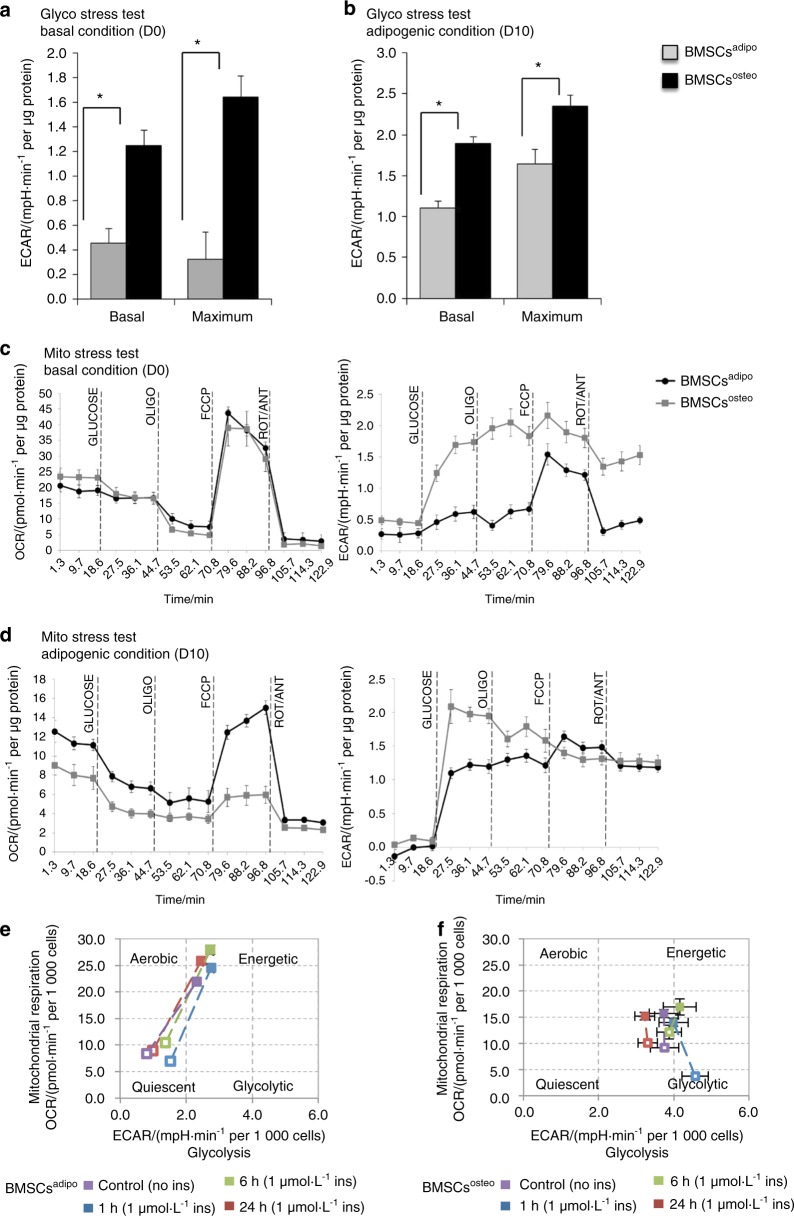
BMSCs^adipo^ and BMSCs^osteo^ exhibit a distinct bioenergetic profile defining their metabolic program. Bioenergetic profiling of BMSCs^adipo^ and BMSCs^osteo^ in basal (D0) and adipogenic conditions (D10) using Seahorse technology: **a**, **b** in the glycolysis stress test measuring glycolytic function in cells; **a** basal and maximal glycolytic capacity in BMSCs^osteo^ compared with capacities in BMSCs^adipo^ in basal conditions (D0) and **b** adipogenic conditions (D10); (*n* = 3) *P < 0.05: BMSCs^adipo^ vs BMSCs^osteo^. **c**, **d** Bioenergetic profiling of undifferentiated and differentiated BMSCs^adipo^ and BMSCs^osteo^ in a Mito stress test measuring mitochondrial respiration in cells. **c** Representative graph of OCR and ECAR in media over time with a treatment of specific molecules (glucose - oligomycin (OLIGO) - carbonyl cyanide-*4*-(trifluoromethoxy)phenylhydrazone (FCCP) - rotenone/antimycin (ROT/ANT) corresponding to vertical line definition) in basal conditions (D0) and **d** adipogenic conditions; (*n* = 3) ^#^*P* < 0.05: BMSCs^adipo^ vs BMSCs^osteo^ under adipogenic conditions (D10), two-tailed unpaired Student’s *t* test. **e** Acute effects of insulin (1 µmol·L^–1^) on the metabolic phenotype of BMSCs^adipo^ and **f** BMSCs^osteo^; (*n* = 3)

Since BMSCs^adipo^ and BMSCs^osteo^ exhibit well-defined differences in their metabolic molecular signature and insulin responsiveness, we investigated the functional consequences on their bioenergetic profile using a Seahorse XF24^®^ analyzer. The cells were studied under the basal state and following incubation in adipogenic culture conditions. We obtained simultaneous measurements of mitochondrial function via the oxygen consumption rate (OCR) and glycolysis via the extracellular acidification rate (ECAR) employing the Glyco and Mito Stress test (Fig. 3).

In the Glyco Stress test, ECAR revealed higher basal glycolytic activity and maximal glycolytic capacity in BMSCs^osteo^ compared with those in BMSCs^adipo^, and this was observed both at baseline (Fig. 3a) and following incubation under adipogenic culture conditions (Fig. 3b). On the other hand, the Mito Stress test showed similar OxPhos activity at baseline between BMSCs^osteo^ and BMSCs^adipo^ (Fig. 3c, left panel). In addition, BMSCs^osteo^ maintained higher glycolytic activity (Fig. 3c, right panel). Following 10 days of incubation under adipogenic culture conditions, BMSCs^adipo^ demonstrated elevated basal OCR, and following mitochondrial uncoupling with carbonyl cyanide-*4*-(trifluoromethoxy) phenylhydrazone (FCCP), their maximal OCR was dramatically increased (Fig. 3d, left panel). Conversely, BMSCs^osteo^ exhibited reduced basal and maximal OCR (mitochondrial respiration) (Fig. 3d, left panel) while maintaining higher glycolytic activities (Fig. 3d, right panel). Furthermore, when BMSCs^adipo^ and BMSCs^osteo^ were exposed to insulin (100 µmol·L^–1^), they exhibited significant differences in their bioenergetic profile with enhanced OxPhos in BMSCs^adipo^ and not in BMSCs^osteo^ that maintained a glycolytic profile (Fig. 3e, f).

These data demonstrate that BMSCs^adipo^ exhibit a distinct bioenergetic profile associated with different strategies for glucose utilization compared with that of BMSCs^osteo^.

### BMSCs^adipo^ and BMSCs^osteo^ exhibit significant differences in their metabolite composition

**Fig. 4:**
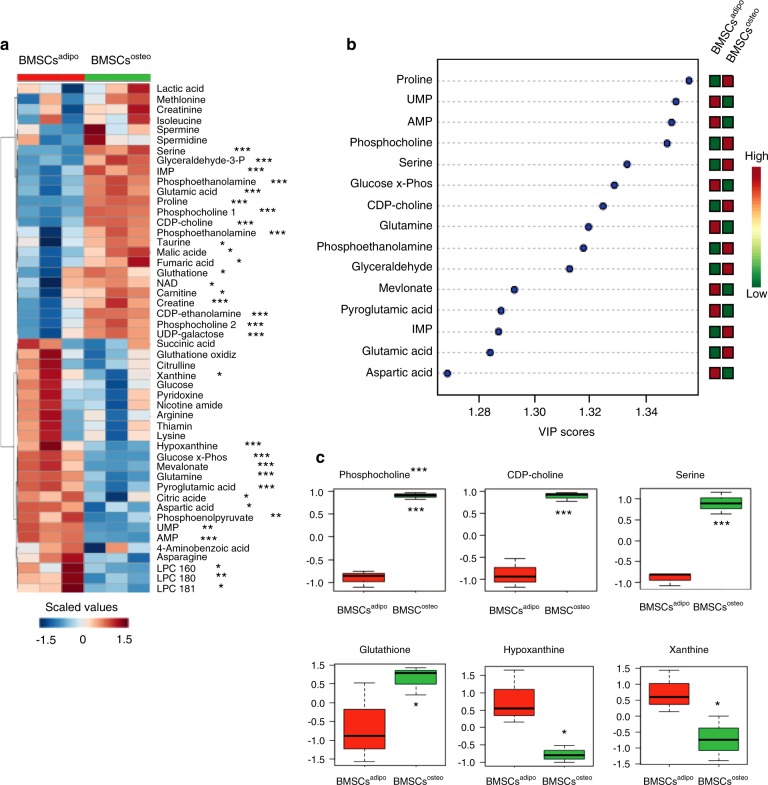
BMSCs^adipo^ and BMSCs^osteo^ exhibit significant differences in their metabolite composition. Global metabolic analyses of intracellular metabolites in basal conditions in BMSCs^adipo^ and BMSCs^osteo^ using liquid chromatography–mass spectrometry (LC–MS). **a** Heat map of all intracellular metabolites that are differentially expressed between BMSCs^adipo^ and BMSCs^osteo^ under basal conditions; **b** important features that are differentially expressed in BMSCs^adipo^ and BMSCs^osteo^; **c** metabolites differentially enriched in BMSCs^adipo^ versus BMSCs^osteo^ such as glycerophospholipids, especially phosphocholine and CPD-choline, glutathione, xanthine, and hypoxanthine. Data are presented as the mean ± SEM from three independent experiments. (*n* = 3) (*P < 0.05, **P < 0.01, ***P < 0.001: BMSCs^adipo^ vs BMSCs^osteo^, two-tailed unpaired Student’s *t* test)

To corroborate the observed changes in the bioenergetic profile, we performed a global metabolomic comparison of BMSCs^adipo^ and BMSCs^osteo^ in undifferentiated conditions using liquid chromatography–mass spectrometry (LC–MS) (Fig. 4). A heat map of intracellular metabolites revealed a unique profile with significant differences detectable at baseline (Fig. 4a). We found that adenosine-5′-monophosphate, guanosine-5′-monophosphate, uridine-5′-monophosphate, glutamine, and glucose-6-phosphate were the major metabolites that distinguish BMSCs^adipo^ from BMSC^sosteo^, suggesting that purine/pyrimidine and glucose metabolism are key processes associated with AD lineage commitment. On the other hand, the major metabolites in BMSCs^osteo^ were proline, choline, glyceraldehyde, and serine, which may be related to the capacity for extracellular matrix production (Fig. 4b).

Additional targeted analysis of metabolites using LC–MS identified several specific compounds, such as phosphocholine, CPD-choline, serine, and glutathione, which were enriched in BMSCs^osteo^ versus BMSCs^adipo^ (Fig. 4c). On the other hand, xanthine and hypoxanthine were more abundant in BMSCs^adipo^ compared with BMSCs^osteo^, suggesting changes in membrane lipid content that affect intracellular signaling and the levels of reactive oxygen species.^24^ Additional metabolites that showed differences between the two immortalized cell lines included glyceraldehyde and glutamic acid, supporting the presence of higher glycolytic activity in BMSCs^osteo^. In addition, amino acid metabolites were different in BMSCs^adipo^ compared with BMSCs^osteo^, e.g., higher glutamine in BMSCs^adipo^, which can serve as an alternative carbon source for OxPhos.^25^

A similar distinct pattern of metabolites was identified in the metabolomic analysis of intracellular metabolites of BMSCs^adipo^ and BMSCs^osteo^ following 24 h and 72 h of in vitro culture in basal conditions (Figs. S4 and S5), corroborating the presence of a stable metabolic program.

### Is the metabolic program of BMSC progenitors flexible? Effects of parathyroid hormone (PTH) and inhibitors of insulin signaling and OxPhos

**Fig. 5:**
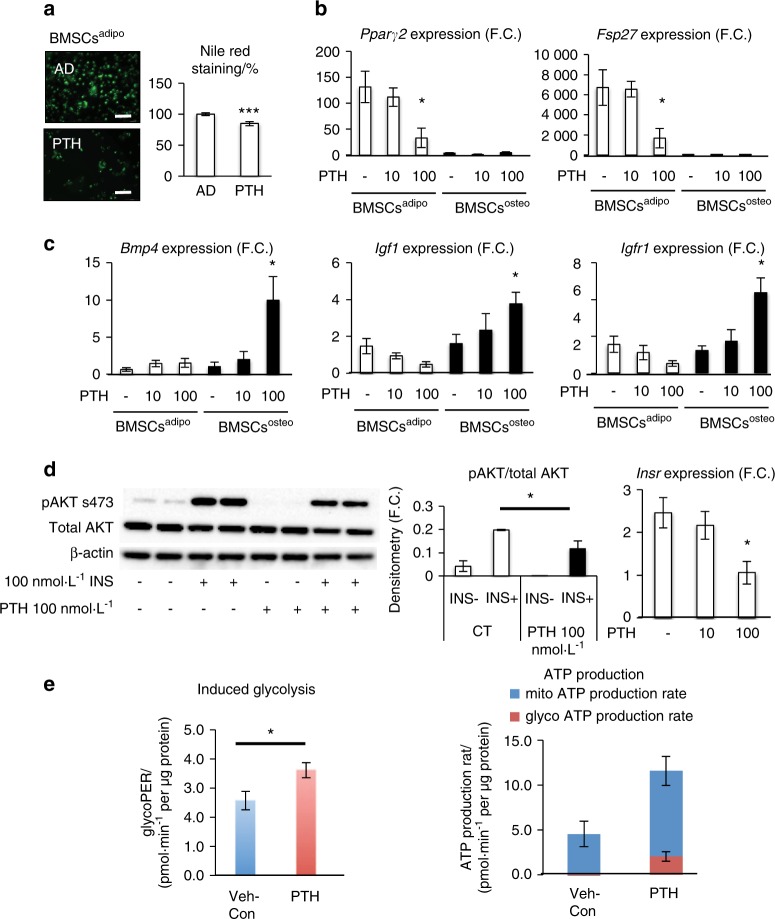
PTH affects the adipogenic potential of BMSCs^adipo^ progenitors. Evaluation of PTH treatment on AD differentiation potential in BMSCs^adipo^ and BMSCs^osteo^. **a** Representative pictures and evaluation of Nile Red staining in BMSCs^adipo^ differentiated in adipogenic conditions (AD) and after chronic (D10) PTH treatment, (scale bar 100 μm); **b** gene expression of adipocytic and insulin signaling-related genes such as *Pparγ2, Fsp27*, and *Irs1* after chronic PTH treatment; **c** gene expression of PTH-responsive genes such as *Bmp4, Igf1*, and *Igfr1* after chronic PTH treatment; data are presented as the mean of the fold change (F.C.) over undifferentiated cells ± SEM, (*n* = 3) **d** representative western blot and densitometry of insulin-stimulated (100 nmol·L^–1^, 15 min) phosphorylation of AKT (p-S473AKT) and total AKT and gene expression of *Insr* in BMSCs^adipo^ treated with PTH 100 nmol·L^–1^; (*n* = 3); **e** glycolytic potential of BMSCs^adipo^ and ATP production after 24 h of PTH treatment (*n* = 3); data are presented as the mean ± SEM (*P < 0.05, **P < 0.01; ***P < 0.001: BMSCs^adipo^ vs BMSCs^osteo^, two-tailed unpaired Student’s *t* test)

Our study demonstrated that committed adipocytic and osteoblastic cells exhibit a distinct metabolic program leading to differential responses under adipogenic culture conditions. However, it is not known whether these responses can be regulated by external cues. Thus, we studied the effects of treatment with PTH on AD differentiation when the cells were cultured under adipogenic culture conditions. PTH is known to enhance OB differentiation of progenitor cells through inducing changes in the bioenergetic profile.^26^ Gene expression profiling revealed that the expression level of PTH receptor 1 (*Pth1r*) was higher in BMSCs^osteo^ compared with the level in BMSCs^adipo^ (Fig. 1k). In vitro treatment of BMSCs^adipo^ and BMSCs^osteo^ with PTH under adipogenic culture conditions for up to 10 days led to reduced AD differentiation in BMSCs^adipo^, as evidenced by the decreased number of Nile Red-positive mature ADs and reduced expression levels of adipocytic genes (*Pparγ2* and *Fsp27*) (Fig. 5a, b). On the other hand, we observed increased expression of the PTH-responsive genes *Bmp4, Igf1*, and *Igfr1* in BMSCs^osteo^ but not in BMSCs^adipo^ (Fig. 5c). Furthermore, PTH treatment impaired insulin signaling accompanied by decreased *Insr* gene expression in BMSCs^adipo^ (Fig. 5d), which corroborates similar findings previously reported in 3T3-Ll cells.^27^ In addition, PTH treatment altered the bioenergetic program of BMSCs^adipo^, shifting the cells towards a more glycolytic state (Fig. 5e**)**, as we observed increased “induced glycolysis” in the presence of PTH (PTH-treated versus Veh-treated cells, 22%, *P* < 0.05) (Fig. 5e, left graph). In addition, PTH treatment induced a shift toward more glycolytic ATP production, even though mitochondrial ATP production was also affected (Fig. 5e, right graph).

**Fig. 6:**
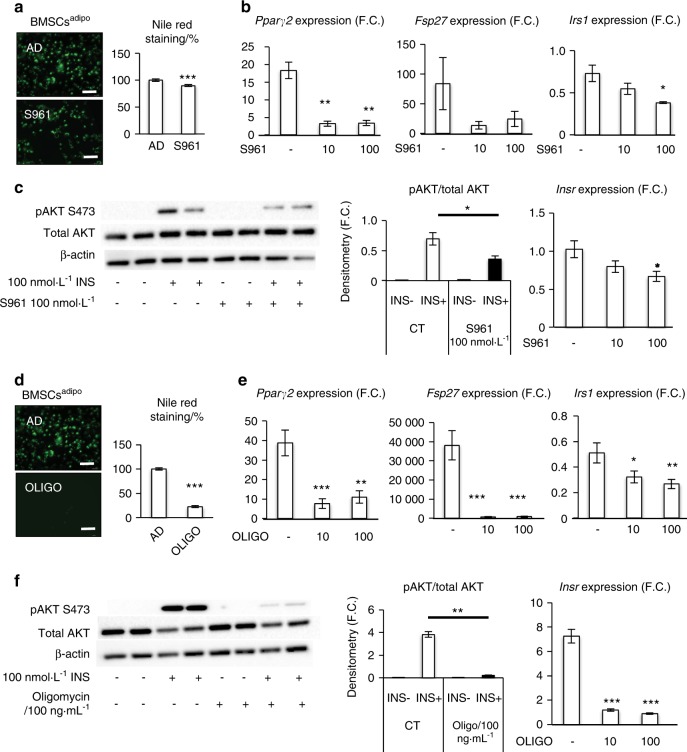
Blocking insulin receptor signaling and oxidative phosphorylation affects the adipogenic potential of BMSCs^adipo^ progenitors. **a** Representative pictures and evaluation of Nile Red staining in BMSCs^adipo^ differentiated in adipogenic conditions (AD) and after 100 nmol·L^–1^ S961 (insulin receptor antagonist) treatment (D10), (scale bar 100 μm); **b** Gene expression of adipocytic and insulin signaling-related genes such as *Pparγ2, Fsp27*, and *Irs1* after S961 treatment; data are presented as the mean of the fold change (F.C.) over undifferentiated cells ± SEM, (*n* = 3); **c** representative western blot and densitometry of insulin-stimulated (100 nmol·L^–1^, 15 min) phosphorylation of AKT (p-S473AKT) and total AKT and gene expression of *Insr* in BMSCs^adipo^ treated with S961 100 nmol·L^–1^; (*n* = 3); **d** representative pictures and evaluation of Nile Red staining in BMSCs^adipo^ differentiated in adipogenic conditions (AD) and after 100 ng·mL^–1^ oligomycin (inhibitor of ATP-synthase) treatment (D7), (scale bar 100 μm); **e** gene expression of adipocytic and insulin signaling-related genes such as *Pparγ2, Fsp27*, and *Irs1* after oligomycin treatment; data are presented as the mean fold change (F.C.) over undifferentiated cells ± SEM, (*n* = 3); **f** representative western blot and densitometry of insulin-stimulated (100 nmol·L^–1^, 15 min) phosphorylation of AKT (p-S473AKT) and total AKT and gene expression of *Insr* in BMSCs^adipo^ treated with oligomycin 10 and 100 ng·mL^–1^; (*n* = 2–3); (*P < 0.05, **P < 0.01; ***P < 0.001: CT vs treated cells; two-tailed unpaired Student’s *t* test)

We also tested whether manipulation of insulin signaling in BMSCs^adipo^ affects their differentiation state. Treatment of BMSCs^adipo^ with the INSR antagonist S961 (100 nmol·L^–1^) under adipogenic culture conditions for 10 days resulted in impaired AD differentiation as evaluated by Nile Red staining (Fig. 6a), reduced gene expression of adipocytic genes (*Pparγ2, Fsp27*, and *Irs1*) (Fig. 6b) and impaired insulin signaling as well as decreased *Insr* gene expression (Fig. 6c). S961 treatment changed basal metabolism in BMSCs^adipo^, as shown by a reduced glycolytic and enhanced OxPhos ATP production rate. In addition, based on ATP levels, S961 treatment increased ATP production in BMSCs^adipo^ to comparable levels as those observed in BMSCs^osteo^ (Fig. S6a). Finally, we tested whether inhibition of OxPhos in BMSCs^adipo^ affects the differentiation capacity of cells. We treated BMSCs^adipo^ with oligomycin (100 nmol·L^–1^) (an inhibitor of ATP-synthase, a key enzyme in mitochondrial respiration) for 10 days in the presence of adipogenic culture conditions. As shown in Fig. 6d, oligomycin treatment decreased the AD differentiation capacity as evidenced by a decreased number of mature ADs that were positive for Nile Red staining, decreased gene expression of adipocytic and insulin signaling-related genes (*Pparγ2, Fsp27*, and *Irs1*) (Fig. 6e) and reduced insulin signaling along with decreased *Insr* gene expression (Fig. 6f). Moreover, oligomycin treatment exhibited modest effects on ATP production in BMSCs^adipo^ under basal undifferentiated culture conditions. However, under adipogenic induction, oligomycin significantly increased ATP production via glycolysis and reduced OxPhos (Fig. S6a, b).

Next, we examined the effect of S961 and oligomycin treatment on OB differentiation. S961 did not exert additional effects on enhanced OB differentiation in BMSCs^osteo^ as determined by in vitro mineralized matrix formation, even though we observed a significant increase in the osteoblastic genes *Alpl* and *Bmp2* (Fig. S6c). Oligomycin significantly inhibited OB differentiation of BMSCs^osteo^, which was also accompanied by inhibition of cell proliferation following longer in vitro exposure (Fig. S6d).

Thus, these data demonstrate that targeting the metabolic program in BMSC progenitors modulates their differentiation capacity.

### In vivo changes in the BM microenvironment induced by a HFD regulate AD and OB progenitor formation

**Fig. 7:**
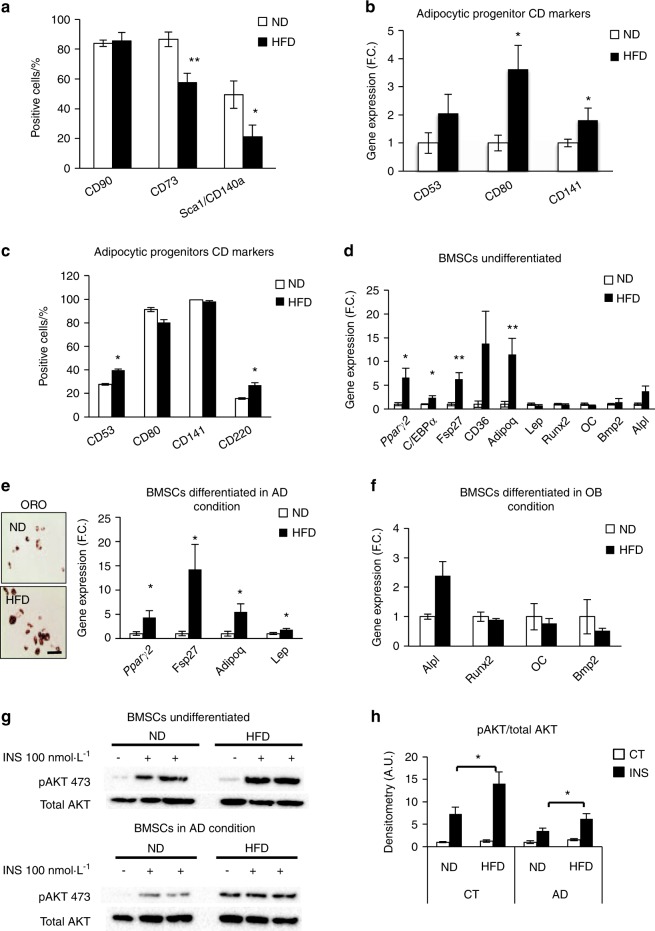
In vivo changes in the bone marrow microenvironment induced by a high-fat diet (HFD) regulate adipocyte and osteoblast progenitors. **a** Screening of stem cell marker expression measured by flow cytometry in primary BMSCs isolated from mice fed with an ND or HFD for 12 weeks (*n* = 5 per group). **b** Gene expression of adipocytic progenitor CD markers (CD53, CD80, and CD141); and **c** flow cytometry of adipocytic CD markers (CD53, CD80, CD141, and CD220) in BMSCs obtained from ND and HFD mice (*n* = 6 per group). Data are presented as the mean of the fold change (F.C.) normalized to ND BMSC gene expression ± SEM; *P < 0.05: ND vs HFD, two-tailed unpaired Student’s *t* test. **d** Gene expression profile of adipocytic genes (*Pparγ2, C/ebpα, Fsp27, CD36, Adipoq*, and *Lep*) and osteoblastic genes (*Runx2, OC, Bmp2*, and *Alpl*) presented as the fold change in BMSCs of ND and HFD mice (*n* = 6 per group); **e** representative pictures of AD differentiation (day 10) visualized with Oil Red O staining in BMSCs from ND and HFD mice, (scale bar 100 μm) and gene expression profile of adipocytic genes (*Pparγ2, Fsp27, Adipoq*, and *Lep*) presented as the fold change in AD differentiated BMSCs of ND and HFD mice (*n* = 6 per group); **f** gene expression profile of osteoblastic genes (*Alpl*, *Runx2, OC*, and *Bmp2)* presented as the fold change in OB differentiated BMSCs of ND and HFD mice (*n* = 6 per group); **g** representative western blot of insulin-stimulated (100 nmol·L^–1^, 15 min) phosphorylation of AKT (p-S473AKT) and total AKT in undifferentiated and AD differentiated BMSCs isolated from ND and HFD mice (*n* = 5–6 per group); **h** densitometry of pAKT/total AKT in undifferentiated and AD differentiated BMSCs isolated from ND and HFD mice. Data are presented as the mean ± SEM; *P < 0.05, **P < 0.01 compared with ND, two-tailed unpaired Student’s *t* test

To determine the physiological relevance of our findings, we employed the molecular signature of BMSCs^adipo^ to investigate the in vivo response of BMSC progenitors to the “obesogenic” environment present in C57BL/6 mice fed for 12 weeks with a HFD (60% fat)^28^ that resulted in obesity, hyperinsulinemia, and increased BM adiposity and AD size in the BM (Fig. S7a–c). In vitro cultures of BMSCs obtained from HFD-fed mice when compared with BMSCs obtained from mice on a normal diet (ND) exhibited a significant decrease in the number of BMSCs that are CD73+ and Sca+/CD140a+ and a concomitant increase in the expression of the adipocytic progenitor markers (mRNA and protein levels) CD80, CD141, CD53, and CD220 (Fig. 7a–c, Fig. S7d), which have previously been reported to be enriched in BMSCs^adipo^ compared with BMSCs^osteo^ ^21^ (Fig. S7e). These findings were accompanied by upregulation of adipocytic gene markers (*Pparγ2, C/ebpα, Fsp27, CD36*, and *Adipoq*) and no changes in osteoblastic gene markers, suggesting expansion of BMSC AD progenitors (Fig. 7d). In addition, the colony forming unit-fibroblast (CFU-f) capacity of primary cultures of BMSCs was decreased in HFD-fed mice (Fig. S7f). Furthermore, exposing BMSCs obtained from HFD mice to adipogenic conditions in vitro led to increased AD differentiation (Fig. 7e), increased expression of adipocytic genes (*Pparγ2, Adipoq, Fsp27*, and *Lep)* and no effect on OB differentiation (Fig. 7f). In addition, similar to our observations in BMSCs^adipo^, cultured BMSCs obtained from HFD mice revealed enhanced responsiveness to insulin compared with that of BMSCs obtained from ND mice (Fig. 7g, h). Thus, these data demonstrate the expansion of a population of adipocytic progenitors in BM following HFD-induced obesity that is similar in phenotype and functional responses to BMSCs^adipo^.

## Discussion

In this study, we demonstrate that BMSC progenitors display a distinct metabolic program that determines their differentiation fate. AD progenitors exhibit enhanced insulin signaling and a higher capacity for OxPhos and lipid storage, whereas OB progenitors exhibit reduced insulin signaling, utilization of glycolytic activity to generate energy and an absence of lipid storage. We also demonstrate that targeting the AD progenitors’ metabolic program can regulate the efficiency of differentiation. The physiological relevance of these findings was corroborated in vivo through an intervention model of HFD-induced obesity in mice that led to expansion and enhanced differentiation of adipocytic progenitors.

Cellular metabolic programing refers to the metabolic processes that not only provide energy for cellular homeostatic functions but also define the cellular phenotype by mediating changes in posttranslational modifications of histones and transcription factors.^9^ Their metabolites could participate in posttranslational modification of the genome by methylation of transcription factor promoters, which has been shown in muscle- or adipose-derived stem cells.^9,29^ Stem cells in different states of commitment have specific bioenergetic needs that are necessary for their functions; thus, their differentiation can be affected under different pathophysiological conditions related to the metabolic status of the organism. Changes in cellular metabolic programming have been reported to occur during the initial phases of somatic cell programming to pluripotent stem cells and during immune cell differentiation.^8,10,11^ However, limited information is available regarding the relationship between the metabolic phenotype and lineage-committed BMSC OB and AD progenitors.

Employing two functionally different murine progenitor cell lines of AD progenitors (BMSCs^adipo^) and OB progenitors (BMSCs^osteo^), we observed a distinct metabolic gene signature of BMSCs^adipo^ characterized by enrichment in genes of insulin signaling, autophagy, lipid metabolism, and glucose utilization, suggesting the presence of a hypermetabolic state. On the other hand, BMSCs^osteo^ were enriched in skeletal-associated genes and extracellular matrix genes that are relevant to their role in matrix production and bone formation. This molecular phenotype suggests that BMSC progenitors mediate their differentiation functions in response to exogenous metabolic cues. These data corroborate recent findings from our group, reporting expansion of adipogenic progenitors in BMSC cultures of obese subjects along with enhanced insulin signaling.^30^

Progenitor cell clonal expansion requires the availability of a quick source of energy, and glycolysis is the preferred energy production mechanism, whereas the more energy efficient oxidative metabolism is associated with cell differentiation and mature functions.^9^ We observed a different metabolic pattern in BMSC progenitors. Committed osteoblastic progenitors employed glycolysis and, to a lesser degree, OxPhos, but committed adipocytic progenitors employed mainly OxPhos. This phenomenon has also been observed in undifferentiated versus differentiated pluripotent stem cells.^31^ The higher glycolytic profile of BMSCs^osteo^ represents a similarity with the stem cell state, while the higher OxPhos rate in BMSCs^adipo^ is associated with a more differentiated phenotype. It is plausible that glycolysis is preferred in committed osteoblastic cells because it is a source of rapid energy generation needed by the cells to respond quickly during bone regeneration following bone fracture or during bone remodeling. In addition, osteoblastic progenitors are present near the bone surface in a hypoxic microenvironment in which HIF1α is inducible and can drive the glycolytic processes.^32,33^ Our study corroborates the findings of a recent study by Guntur et al.^34^ demonstrating that glycolysis is a major metabolic process during OB differentiation in a murine osteoblastic cell line MC3T3, in contrast to a committed preadipocytic cell line, 3T3-L1, which preferred OxPhos during AD differentiation. Thus, commitment to either OBs or ADs is associated with a characteristic bioenergetic profile that is maintained during differentiation.

Nutrient-sensitive signaling pathways (mTOR, AMPK, or insulin/IGF-1) contribute to stem cell fate determination and energy fuel choice.^35^ Glucose is transported into the cells via glucose transporters (GLUT) that vary among different cell types.^36^ During skeletal development, glucose uptake is mediated primarily via GLUT1, which is insulin-independent.^37^ In our study, we observed that postnatal BM AD BMSCs^adipo^ progenitors expressed higher levels of insulin-responsive GLUT4 compared with BMSCs^osteo^, suggesting a higher responsiveness to insulin. This was further confirmed by the presence of enhanced insulin-stimulated glucose uptake in AD progenitors and significant changes in the metabolic phenotype in response to the acute effect of insulin when compared with BMSCs^osteo^. Our data corroborate and extend the results of Li et al.^38^ that different GLUT are utilized by murine bone cells depending on their developmental stage.

Is it possible to change lineage fate through targeting the metabolic program of BMSC committed progenitors? We observed that PTH treatment, inhibitors of insulin signaling and OxPhos affected the cellular metabolism of AD progenitors and inhibited their differentiation. Recent studies support the observed effects in our study. Fan et al.^26^ demonstrated that PTH/PTH1r signaling regulates BMSC cell fate and enhances OB lineage commitment. Similar to our findings, Esen et al. showed that PTH promotes OB differentiation by enhancing glycolysis,^39^ and Maridas et al. reported^40^ PTH-enhancing effects on BMAT lipolysis. Here, we also presented additional evidence that targeting insulin signaling or OxPhos can influence the differentiation fate of BMSC progenitors. While targeting insulin signaling in BMSC progenitors showed an opposite effect on AD and OB differentiation, inhibition of OxPhos by a long exposure of oligomycin negatively affected both BMSCs^adipo^ and BMSCs^osteo^ progenitors during differentiation. However, further studies are needed to investigate whether acute treatment with oligomycin exhibits a similar effect. As previously shown by Kim et al.^41^ and Guntur et al.,^34^ OBs may use OxPhos during their differentiation, and depending on the source of fuel, either FA or glucose, this was stimulated or inhibited.

Our findings suggest that BMSC progenitors exhibit a metabolic program upstream of differentiation and not, as commonly assumed, that as progenitors differentiate, they acquire changes in their metabolic program. This difference is important in clarifying the role of BM ADs in whole body energy metabolism and bone homeostasis.

An inverse relationship between BMAT and bone mass has been observed in several studies. However, analysis of the published data shows that in many situations, BMAT and bone mass can change independently.^7^ Our study provides a cellular explanation for these differences, as it demonstrates the presence of AD and OB progenitors within the BM microenvironment that respond differently to external stimuli as well as to the metabolic status of the organism. In hyperinsulinemic, hyperglycemic, and hyperlipidemic conditions using HFD-fed mice, we and others^42^ showed expansion of adipocytic progenitors in the BM microenvironment. In addition, our data advance the concept that osteoblastic and adipocytic mature cell formation can be regulated not only at the level of stem cells but also at the level of committed progenitors. For translational medicine, manipulation of metabolic pathways in BMSC progenitors is a plausible approach to enhance lineage-specific differentiation and tissue regeneration.

## Materials and methods

### Cell culture of MSC clonal cell lines (BMSCs^adipo^ and BMSCs^osteo^)

The murine BM-derived spontaneously immortalized clonal cell lines BMSCs^adipo^ and BMSCs^osteo^ were established and characterized previously in our laboratory (for more information, see papers^19,20^). For expansion, both cell lines were cultivated in basal DMEM supplemented with 10% fetal bovine serum (FBS; Gibco Invitrogen, USA) and 100 μg·mL^–1^ streptomycin and 100 U·mL^–1^ penicillin.

### Microarray analyses

BMSCs^adipo^ and BMSCs^osteo^ were cultured under standard culture conditions as described above. Total RNA was isolated at day 0 to perform global gene expression analysis using Affymetrix GeneChip® MG430A 2.0 Arrays (Affymetrix, USA) (GEO accession number: GSE124905). These data were published in a previous publication by Taipaleenmäki et al.^20^ We performed addition analyses to compare global gene expression in basal conditions to determine the GO categories of the genes that were differentially expressed between BMSCs^adipo^ and BMSCs^osteo^ (Fig. 1b and Fig. S1).

### AD differentiation

For in vitro AD differentiation, the cells from both cell lines were seeded at a density of 20 000 cells·cm^–2^ and incubated in DMEM supplemented with 9% horse serum and ADinduction medium containing 3 μg·mL^–1^ insulin (Sigma), 1 × 10^−6^ mol·L^–1^ dexamethasone, and 0.5 μmol·L^–1^ 3-isobutyl-1-methylxanthine (IBMX; Sigma). The medium was changed every second day for 10 days. The ADs were visualized by Oil Red O staining.

### Oil Red O staining

Cells were fixed in 4% paraformaldehyde for 10 min at room temperature (RT) and then stained with Oil Red O (Sigma-Aldrich) to visualize the lipid content. Briefly, cells were rinsed in 3% isopropanol solution and stained with filtered Oil Red O solution (0.5 g in 100% isopropanol) for 1 h at RT.

### Nile red staining

Nile red fluorescence quantification of adipogenesis was performed using a stock solution of Nile red (1 mg·mL^–1^) in DMSO. Staining was performed on unfixed cells. Cultured differentiated cells were grown in polystyrene flat‐bottom 96‐well tissue culture (TC)‐treated black microplates and washed once with PBS. The dye was directly added to the cells (5 μg·mL^–1^ in PBS) and incubated for 10 min at RT, and then the cells were washed twice with PBS. The fluorescence intensity was measured using a microplate reader with the bottom well‐scan mode, during which nine readings were taken per well using excitation (485 nm) and emission (572 nm) spectra.

### OB differentiation

The cells were plated at a density of 20 000 cells·cm^–2^ in alpha MEM medium (Gibco) containing 10% FBS, 100 U·mL^–1^ penicillin (Gibco), 100 g·mL^–1^ streptomycin (Gibco). One day after seeding, the medium was replaced with OB induction medium composed of base medium supplemented with 10 mmol·L^–1^ B-glycerophosphate (Sigma-Aldrich), 10 nmol·L^–1^ dexamethasone (Sigma-Aldrich) and 50 μg·mL^–1^ vitamin C (Sigma-Aldrich). The medium was changed every other day for 10 days.

### ALP activity assay

Cells were incubated with naphthol AS-TR phosphate solution containing Fast Red TR (Sigma-Aldrich) as described previously.^43^ ALP activity was measured using p-nitrophenyl phosphate (Fluka Chemie) as the substrate.^44^

### Quantitative real-time PCR (qRT-PCR)

Total RNA was extracted from the cells using TRIzol (Invitrogen) following the manufacturer’s protocol. The RNA concentration was measured using a Nanodrop 2100, and cDNA synthesis was performed from 1 μg of total RNA using a commercial revertAid H minus first strand cDNA synthesis kit (Fermentas, Helsingborg, Sweden) according to the manufacturer’s instructions. Gene expression was analyzed by qRT-PCR using IQ SYBR Green Master Mix with the ABI Step One Plus Real-Time PCR System (Applied Biosystems) according to the manufacturer’s instructions. A list of primers used is given in Table S1. qPCR data were normalized to the housekeeping gene 36B4 expression and calculated using the formula 2^−(ΔΔCt)^ in which ΔΔCt = (Ct mRNA − Ct 36B4)_sample_ − (Ct mRNA − Ct 36B4)_control sample_.

### Western blot analyses

Cell lysates were prepared using RIPA lysis buffer. Protein concentrations were measured using the BCA assay (Thermo Fisher). Thirty micrograms of protein was separated on 8%–12% NuPAGE Novex Bis-Tris gels (Invitrogen) followed by transfer to a polyvinylidene fluoride membrane (Millipore A/S). Antibodies against the INSR and total or Ser-473 phosphorylated AKT (pAKT) were obtained from Cell Signaling Technology (Herlev, Denmark). Anti-β-actin was purchased from Santa Cruz Biotechnology, Inc. (Aarhus, Denmark). Quantification of western blots was performed with Bio-Rad software.

### Glucose uptake

Glucose uptake was measured using [1,2-3H]−2-deoxy-D-glucose as previously described.^45^ The cells were starved 4 h before the assay in basal medium without serum (DMEM/0.5% BSA). Then, the cells were incubated in basal DMEM/0.5% BSA containing [1,2-3H]-2-deoxy-D-glucose in the presence or absence of 100 nmol·L^–1^ insulin (Sigma) for 4 h and 24 h. Next, the cells were washed and scraped in PBS buffer, and neutral lipids were separated by TLC. The scintillation counts in cell lysates were measured on a scintillator (PerkinElmer). The results were normalized to protein concentrations and presented as a fold change ± SEM.

### Lipid extraction and TLC

Lipids were extracted using Bligh and Dyer extraction of total lipids from mammalian cells by adding chloroform:methanol (1:1) solution followed by a solution of chloroform:0.2 mol·L^–1^ KCl, which resulted in phase separation of the organic and aqueous phases. The organic phase was removed and dried under nitrogen and then resuspended in chloroform:methanol. A 20-µL aliquot was used for scintillation measurements. The rest of the samples were loaded in duplicate, and TLC plates were developed to the top of the plate in the solvent system hexane:diethylether:acetic acid (80:30:1). Lipids were visualized after spraying the plate with 10% CuSO_4_ dissolved in 8% H_2_PO_4_ and incubating in a 180 °C oven. For radioactive lipid spot visualization, the plates were placed in cassettes (storage radioactive screen) for 1 month in the dark and then the storage screen was scanned on The Typhoon Scanner. Representative scan pictures are presented as a PDF.

### Bioenergetic analyses/profiling

BMSCs^adipo^ and BMSCs^osteo^ murine cell lines were seeded in 24-well multiwell plates (Seahorse Bioscience) and differentiated as described above. OCRs of nondifferentiated and 10-day-differentiated cells were determined using an XF24 Extracellular Flux Analyzer (Seahorse Bioscience). Glycolytic stress tests were performed by sequentially adding 10 mmol·L^–1^ glucose (and 100 nmol·L^–1^ insulin), rotenone and antimycin A (Rot/Ant; 1 µmol·L^–1^ each), carbonyl cyanide-4-(trifuoromethoxy)phenylhydrazone (FCCP; 2 µmol·L^–1^), followed by 2-deoxyglucose (50 mmol·L^–1^). Conversely, a mitochondrial stress test was performed by adding 10 mmol·L^–1^ glucose (and 100 nmol·L^–1^ insulin), oligomycin A (Oligo; 2 µmol·L^–1^), FCCP (2 µmol·L^–1^), and Rot/Ant (1 µmol·L^–1^ each) in succession. The basal OCR was then calculated by subtracting Rot/Ant from the unstimulated OCR value. Maximal respiration was determined following FCCP treatment according to Wu et al.^46^ The ATP production rate was measured by an ATP rate assay, and the acute effects of insulin were measured by an energy phenotype test (Agilent Technologies). Data are presented as normalized to µg protein.

### Metabolomics measured by LC–MC

Sample analysis was carried out by MS-Omics as follows. Intracellular extracts were dried under nitrogen flow and reconstituted in a MilliQ water/acetonitrile (1:1) mixture. Extracellular supernatants were diluted 1:3 in MilliQ water before acetonitrile was added (1:1). The analysis was carried out using a UPLC system (UPLC Acquity, Waters) coupled with a time of flight mass spectrometer (Xevo G2 Tof, Waters). An electrospray ionization interface was used as the ionization source. Analysis was performed in negative and positive ionization mode. The UPLC was performed using a slightly modified version of the protocol described by Paglia et al.^47^ Data processing was carried out using MZmine 2^48^ followed by curation using a custom made in-house protocol. The compounds were identified using both peak retention times (compared with authentic standards) and accurate mass (with an accepted deviation of 0.005 Da). Metabolomic data were analyzed using MetaboAnalyst software (www.metaboanalyst.ca).

### Treatment of BMSC progenitors with S961, PTH, and oligomycin

BMSCs^adipo^ and BMSCs^osteo^ were treated during their adipogenic differentiation protocol with different reagents to test their effect on differentiation: INSR antagonist 10 nmol·L^–1^ or 100 nmol·L^–1^ to block insulin signaling as one of the differently activated signaling pathways between cell lines (S961; cat. no. ABIN2876379) (www.antibodies-online.com), PTH known to enhance OB differentiation (1-34 fragment bovine) (Sigma; cat. no. P3671) 10 nmol·L^–1^ or 100 nmol·L^–1^, and oligomycin 10 nmol·L^–1^ or 100 nmol·L^–1^, an inhibitor of OxPhos (Sigma, cat. no. 75351) (in independent experiments) for 10 days. The AD differentiated cells were harvested after 10 days for subsequent analyses. The treatments with PTH, S961 and oligomycin were tested on mouse BMSCs (mBMSCs) (Fig. S8).

### Animal work

Male C57BL/6J mice (Taconic) were given ad libitum normal chow diet (Altromin® 132003; containing 6% fat, 30% protein, 63% carbohydrate, and 7.7% sucrose) or 60 kcal% HFD (Research Diet D12492; containing 35% fat, 26% protein, 26% carbohydrate, and 8.8% sucrose) at 8 weeks of age for a period of 12 weeks. Animals were bred and housed under standard conditions (21 °C, 55% relative humidity) on a 12-h light/12-h dark cycle. All experimental procedures were approved by the Danish Animal Ethical Committee (2017-15-0201-01210).

### Glucose metabolic studies

A glucose tolerance test (GTT) was performed after 12 weeks of HFD. For GTT, overnight-fasted mice were injected with 1 g·kg^–1^ d-glucose i.p. and glucose levels were measured at different timepoints using a BREEZE® 2 meter (Bayer).

### Isolation of BMSCs

BMSCs were isolated from the bones of the front and hind limbs of C57BL/6J male mice (after 12 weeks of HFD) according to the paper by Tencerova et al.^28^ following the steps of bone crushing, collagenase digestion, and negative selection of CD45, CD31, and Ter119 cells using microbeads (Miltenyi) according to the protocol of Zhu et al. and Houlihan et al.^49,50^ Isolated BMSCs were cultured in vitro, passaged, and used for subsequent analyses.

### CFU assay

For assessment of CFUs, cells were plated at a density of 500 cells in a 60-mm culture dish. After 14 days, colonies displaying more than 50 cells were counted using crystal violet staining (Sigma-Aldrich).

### Flow cytometry

Immunophenotyping of BMSCs was performed by flow cytometry. Adherent cells were removed from flasks using 0.05% trypsin EDTA and incubated with Fc-mouse blocking reagent followed by preconjugated antibodies for isotype controls and specific markers (rat anti-mouse Sca-1-PE, cat. no. 553336; rat anti-mouse CD140a-APC, cat. no. 562777; rat anti-mouse CD73-PE, cat. no. 550741; BD Biosciences and rat anti-mouse CD105-PE, cat. no. 120408; rat anti-mouse CD90.2-PE, cat. no. 140307; rat anti-mouse CD44-PE, cat. no. 103024; BioLegend; rat anti-mouse CD53-FITC, cat. no. 124705, BioLegend; Armenian hamster anti-mouse CD80 BV421, cat. no. 104725; BioLegend; rat anti-mouse CD134-APC, cat. no. 119413, BioLegend; rat anti-mouse CD141-PE, cat. no. 566338, BD Bioscience, goat anti-mouse CD220-PE, cat. no. FAB1544P, R&D Biosystems) used according to the manufacturers’ recommendations. The flow cytometry was performed by a BD FACSAria II (BD Biosciences) and analyzed by FlowLogic analysis software.

### Statistical analysis

The statistical significance of the differences in the means of the investigated cell lines was determined by unpaired Student’s *t* test or one-way ANOVA where appropriate using GraphPad Prism v 6.0c software. For all tests, *P* ≤ 0.05 was considered significant. The data are presented as the means ± SEM.

## Supplementary information

Revised Supplementary information
