# Supplementary material for: Metabolic programming determines the lineage-differentiation fate of murine bone marrow stromal progenitor cells
Source: Bone Res. 2019 Nov 14;7:35. doi: 10.1038/s41413-019-0076-5 (PMC6856123; doi:10.1038/s41413-019-0076-5)
Supplement: Supplementary file 1 — Revised Supplementary information [file 41413_2019_76_MOESM1_ESM.pdf]

SUPPLEMENTARY INFORMATION

Supplementary Figures with figure legends: Fig.S1-S8

Supplementary Table: Table S1

Fig. S1

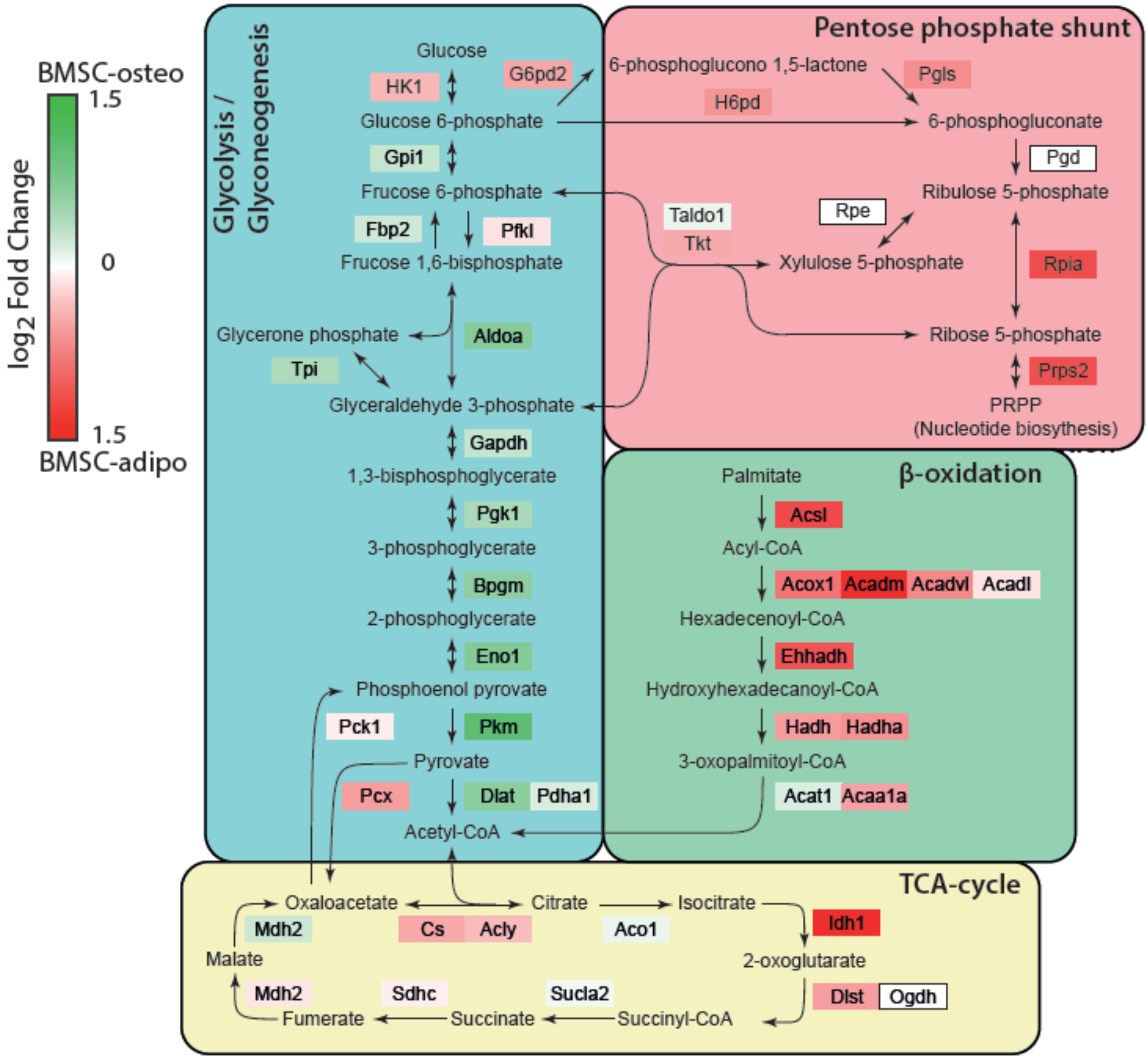

Fig. S1. **Representative scheme of genes involved in metabolic pathways enriched from microarray data.**

Fig. S2

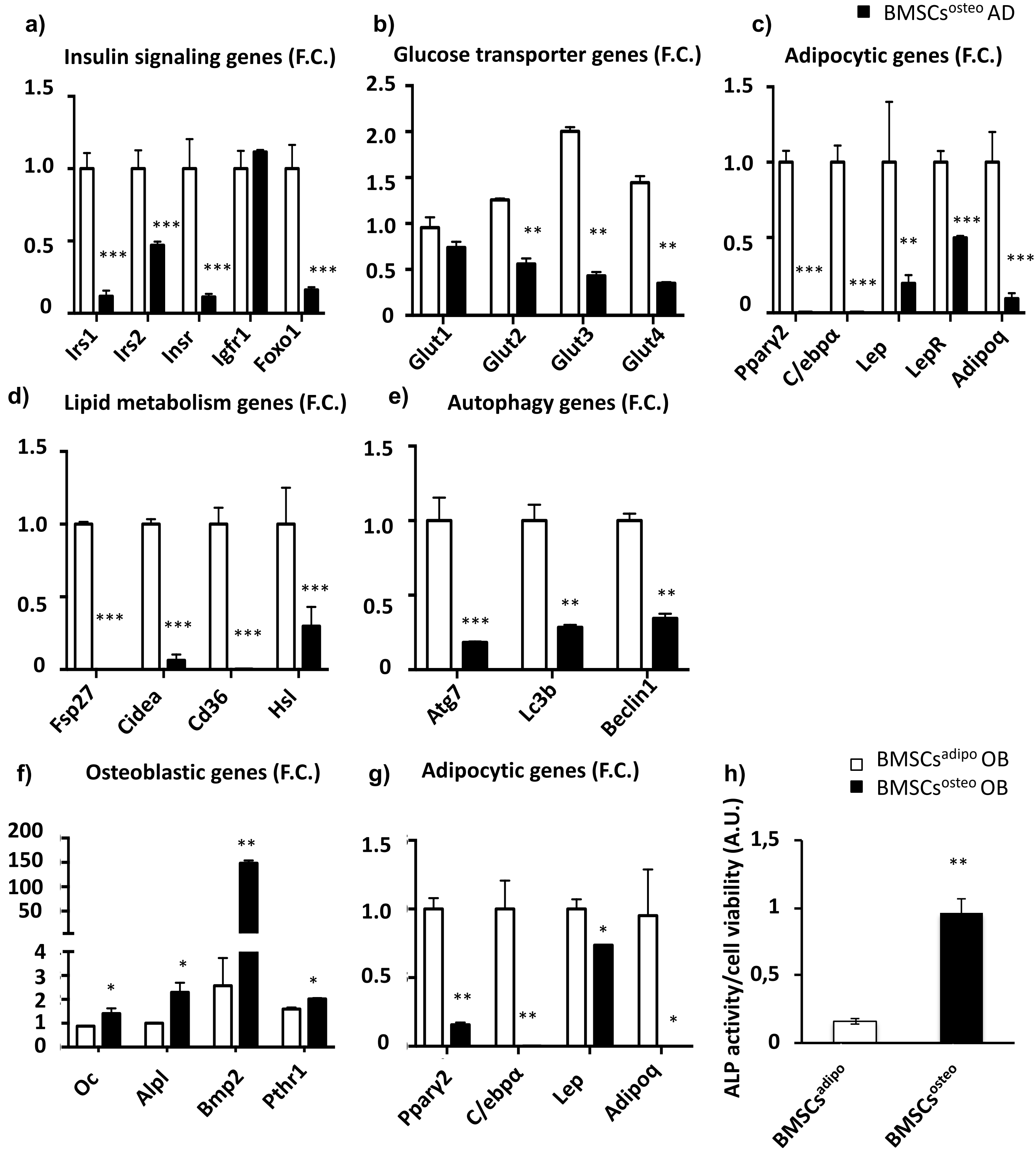

Fig. S2: Gene expression profiling of BMSCs^{adipo} and BMSCs^{osteo} in adipogenic condition (D10) (a-e). (**a**) Genes involved in insulin signaling as *Irs1, Irs2, Insr, Igfr1, Foxo1*; (**b**) glucose transporters as *Glut1, Glut2, Glut3 and Glut4*; (**c**) adipocytic genes as *Pparg2, C/ebpα, Lep, LepR and Adipoq*; (**d**) genes involved in lipid metabolism as *Fsp27, Cidea, CD36, Hsl,* (**e**) autophagy genes as *Atg7, Lc3b,* and *Beclin1*. (**f-g**) Gene expression profiling of BMSCs^{adipo} and BMSCs^{osteo} in osteogenic condition (D10). (**f**) Osteoblastic genes as *Oc, Alpl, Bmp2, Pthr1*; (**g**) Adipocytic genes as *Pparγ2, C/ebpα, Lep, and Adipoq*; (**h**) ALP activity normalized to cell viability of BMSCs^{adipo} and BMSCs^{osteo} in osteogenic condition. Data are presented as mean fold change (F.C.) of gene expression normalized to BMSC^{adipo} expression $\pm$ SEM, (n=3 per group); (*$p<0.05$, **$p<0.01$; ***$p<0.001$: BMSCs^{adipo} vs BMSCs^{osteo}, two-tailed unpaired Student's t test).

Fig. S3

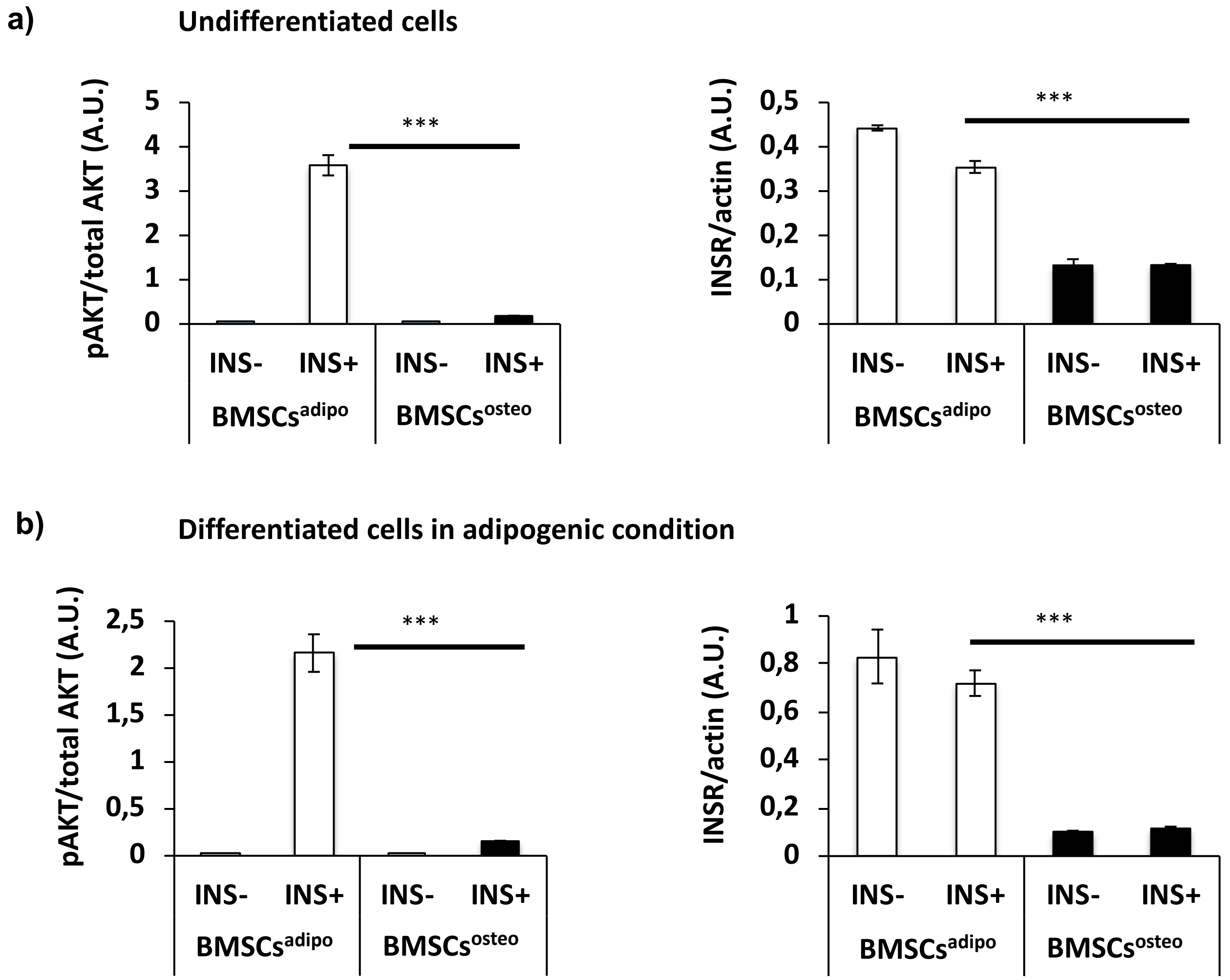

Fig. S3: Densitometry of pAKT/totalAKT and INSR/actin in BMSCs^{adipo} and BMSCs^{osteo}. (a) in undifferentiated cells and **(b)** in adipogenic condition. Data are presented as mean of densitometry ± SEM, (n=3); (***p< 0.001: BMSCs^{adipo} vs BMSCs^{osteo}, two-tailed unpaired Student's t test).

Fig. S4

24h timepoint

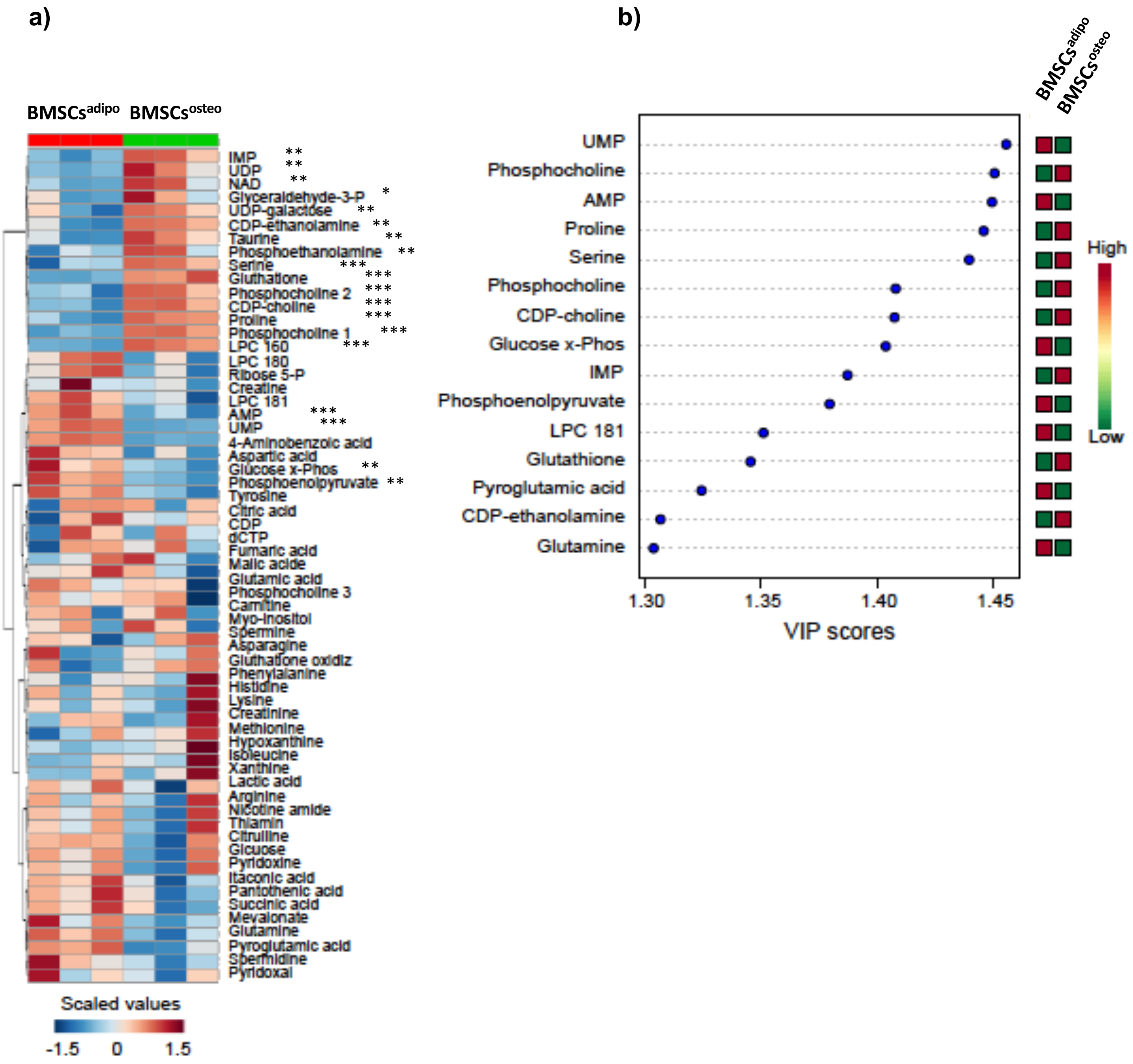

Fig. S4: Global metabolic analyses of intracellular metabolites after 24h in basal medium in BMSCs^{adipo} and BMSCs^{osteo} using LC-MS. (**a**) Heatmap of all intracellular metabolites differently expressed between BMSCs^{adipo} and BMSCs^{osteo} in basal condition after 24h; (**b**) Important features differently expressed in BMSCs^{adipo} and BMSCs^{osteo}. Data are presented as mean ± SEM from three independent experiments. (n= 3) (*p<0.05, **p<0.01, ***p<0.001: BMSCs^{adipo} vs BMSCs^{osteo}, two-tailed unpaired Student's t test).

Fig. S5

72h timepoint

a)

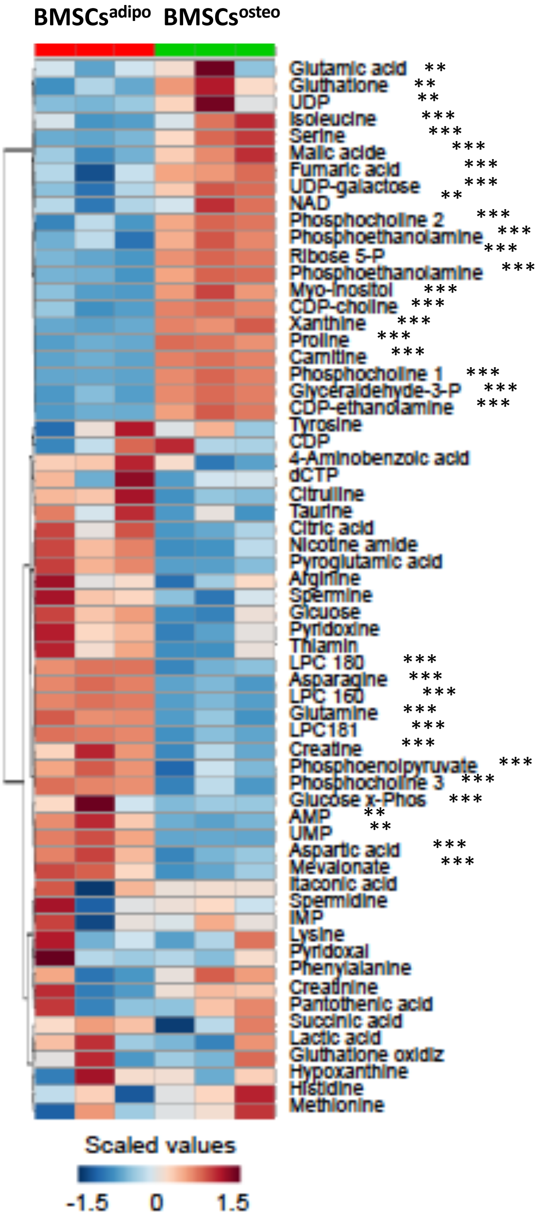

b)

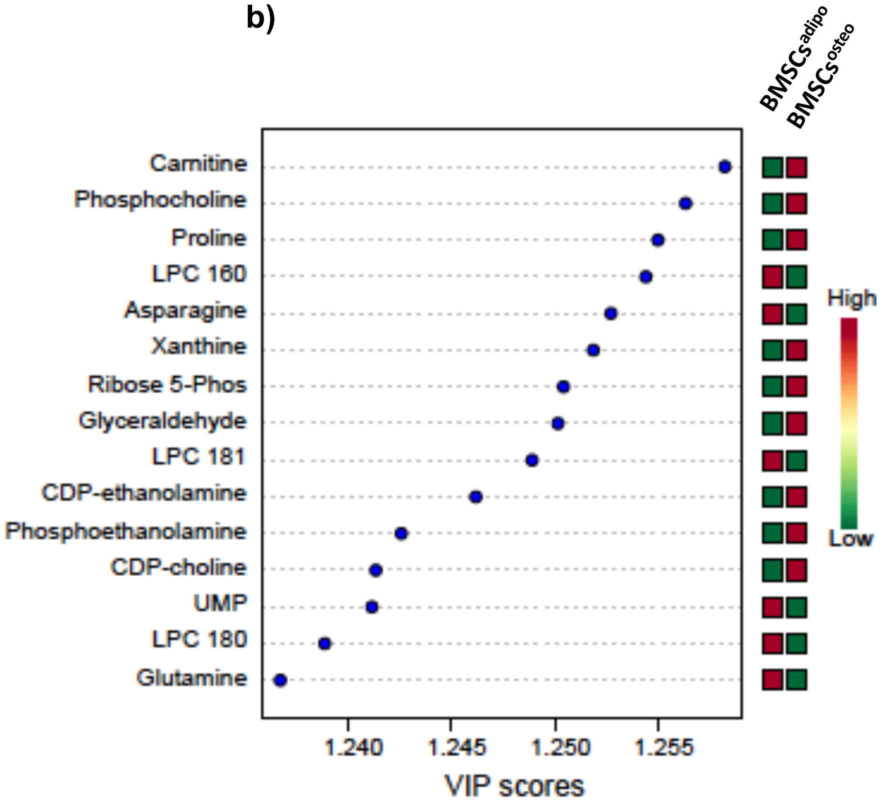

Fig. S5: Global metabolic analyses of intracellular metabolites after 72h in basal medium in BMSCs^{adipo} and BMSCs^{osteo} using LC-MS. (**a**) Heatmap of all intracellular metabolites differently expressed between BMSCs^{adipo} and BMSCs^{osteo} in basal condition after 72h; (**b**) Important features differently expressed in BMSCs^{adipo} and BMSCs^{osteo}. Data are presented as mean ± SEM from three independent experiments. (n= 3) (*p<0.05, **p<0.01, ***p<0.001: BMSCs^{adipo} vs BMSCs^{osteo}, two-tailed unpaired Student's t test).

Fig. S6

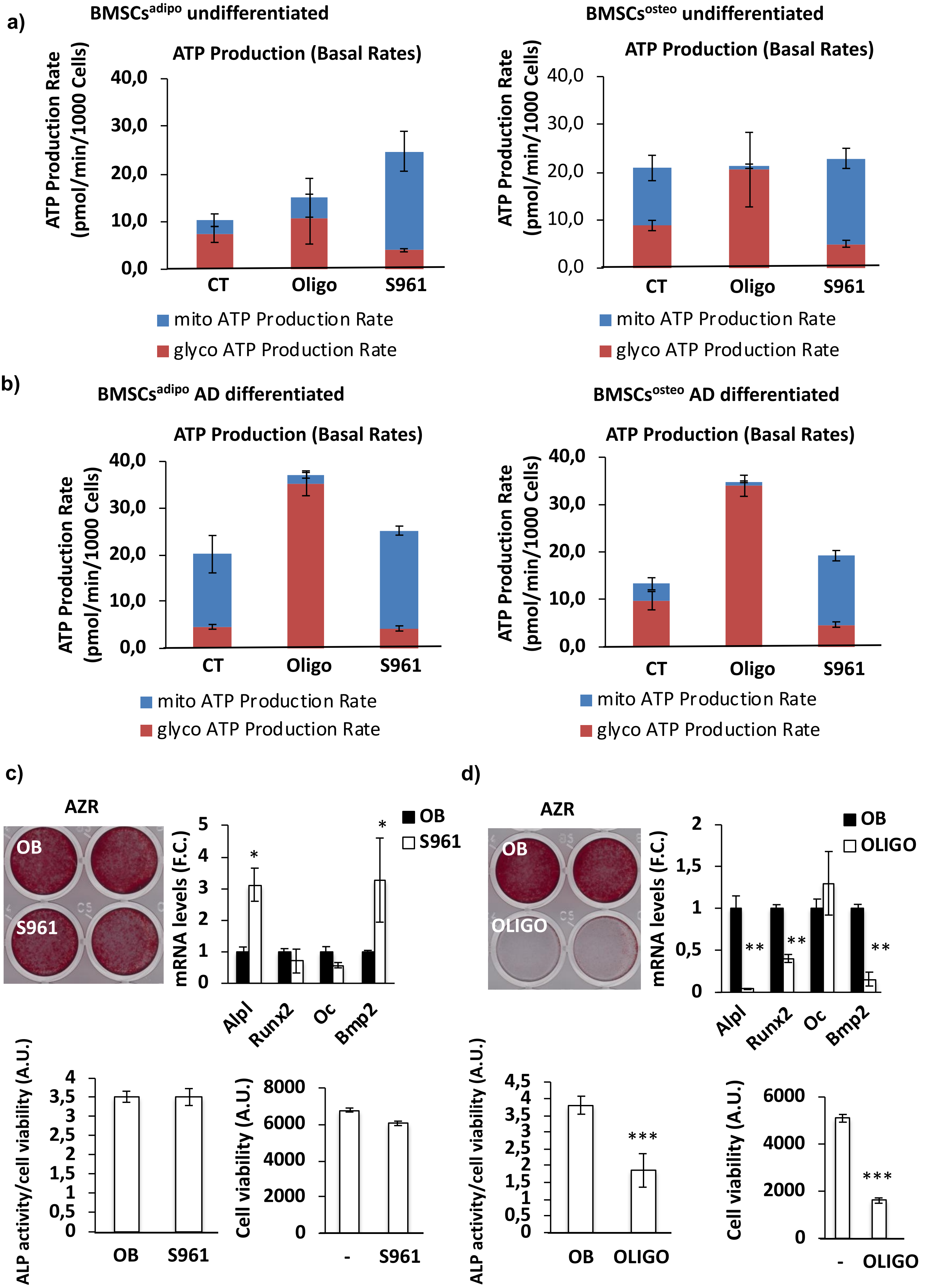
a)
BMSCs adipo undifferentiated
ATP Production (Basal Rates)
ATP Production Rate (pmol/min/1000 Cells)
40,0
30,0
20,0
10,0
0,0
CT
Oligo
S961
mito ATP Production Rate
glyco ATP Production Rate
BMSCs osteo undifferentiated
ATP Production (Basal Rates)
ATP Production Rate (pmol/min/1000 Cells)
CT
Oligo
S961
mito ATP Production Rate
glyco ATP Production Rate
b)
BMSCs adipo AD differentiated
ATP Production (Basal Rates)
ATP Production Rate (pmol/min/1000 Cells)
CT
Oligo
S961
mito ATP Production Rate
glyco ATP Production Rate
BMSCs osteo AD differentiated
ATP Production (Basal Rates)
ATP Production Rate (pmol/min/1000 Cells)
CT
Oligo
S961
mito ATP Production Rate
glyco ATP Production Rate
c)
AZR
OB
S961
mRNA levels (F.C.)
OB
S961
*
*
Alpl
Runx2
Oc
Bmp2
ALP activity/cell viability (A.U.)
OB
S961
Cell viability (A.U.)
-
S961
d)
AZR
OB
OLIGO
mRNA levels (F.C.)
OB
OLIGO
**
**
**
Alpl
Runx2
Oc
Bmp2
ALP activity/cell viability (A.U.)
***
OB
OLIGO
Cell viability (A.U.)
***
-
OLIGO

Fig. S6: Metabolic flexibility of BMSCs^{adipo} and BMSCs^{osteo} and osteoblast differentiation under S961 and Oligomycin treatment. (**a**) ATP production rate in mito vs glycolytic process in undifferentiated BMSCs^{adipo} and BMSCs^{osteo} and (**b**) in AD differentiation (n=3). (**c**) Osteoblast differentiation of BMSCs^{osteo} under S961 treatment measured by Alizarin (AZR) staining, ALP activity normalized to cell viability and gene expression of osteoblastic genes (*Alpl, Runx2, Oc, Bmp2*). (**d**) Osteoblast differentiation of BMSCs^{osteo} under Oligomycin treatment measured by Alizarin (AZR) staining, ALP activity normalized to cell viability and gene expression of osteoblastic genes (*Alpl, Runx2, Oc, Bmp2*). Data are presented as mean ± SEM (n= 2-3 per condition); *p<0,05, **p<0,01 ***p<0,001, two tailed unpaired Student´s t test.

Fig. S7

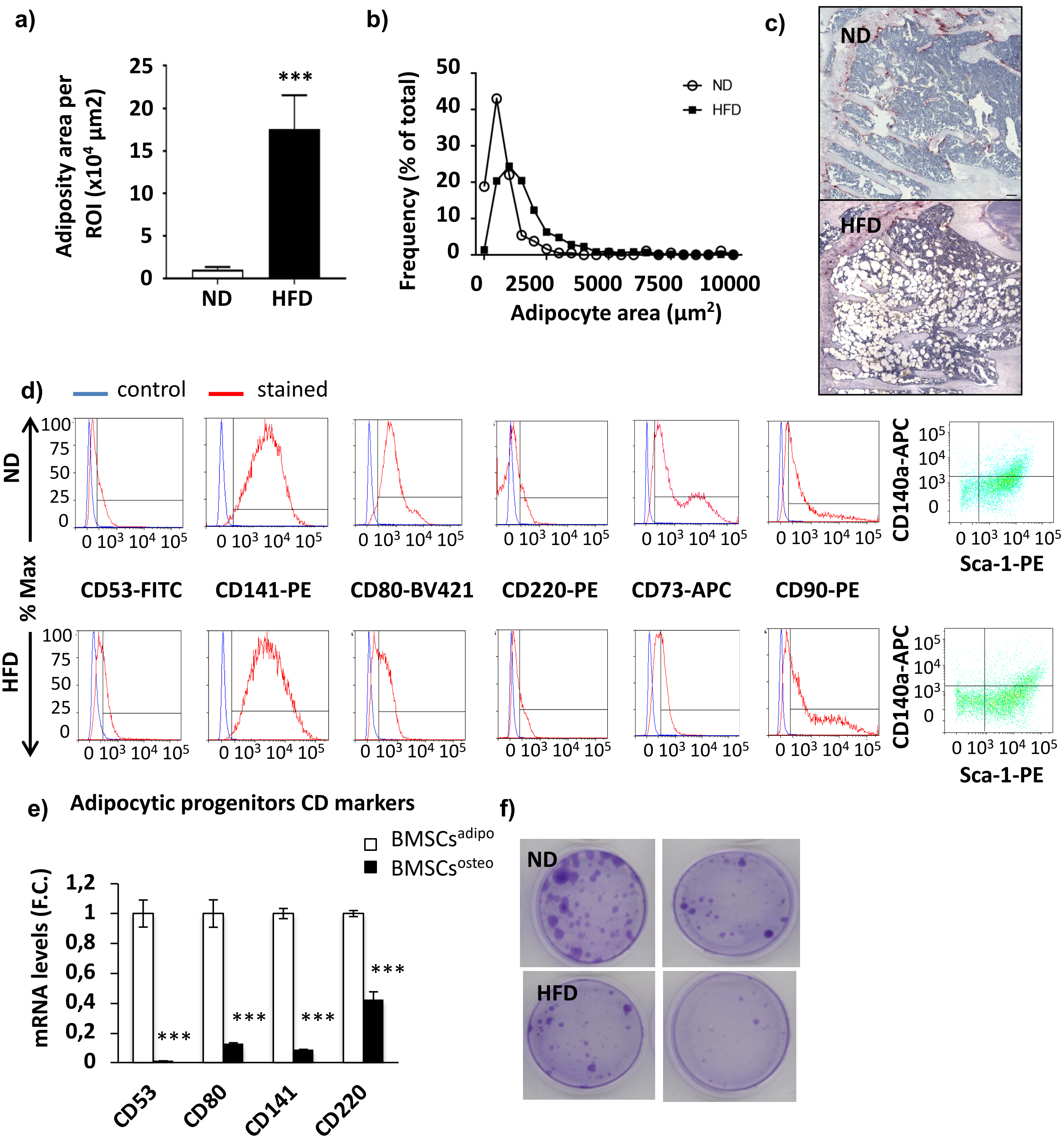

Fig. S7: Bone marrow adiposity evaluation in HFD mice and stem cell properties of BMSC in ND and HFD mice. (**a**) Adiposity area (adipocytes surface per ROI) on TRAP sections. (3 depths per animal). (**b**) The distribution of adipocytes within the bone marrow at the proximal tibia region is calculated using the frequency function of graph pad prism. ND have a mean adipocyte area of 1036.47±123.27 µm^2 and a median of 552.146 µm^2 while HFD have a mean of 1777.84±62.45 µm^2 with a median of 13337.47 µm^2. (**c**) TRAP staining of histological sections of tibia from mice fed for 12 weeks with ND or HFD; scale bar=100µm. Data are presented as mean ±SEM (n=6 per group); ***p<0,001 ND versus HFD, two tailed unpaired Student´s t test. (**d**) Representative histograms and dot plots of stem cell markers in BMSC obtained from ND and HFD mice. (**e**) Gene expression of adipocytic progenitor CD markers in BMSCs^{adipo} and BMSCs^{osteo} (CD53, CD80, CD141, CD220) (n=3); ***p<0,001: BMSCs^{adipo} vs BMSCs^{osteo}. (**f**) Colony forming unit properties in primary cultures of BMSC obtained from ND and HFD mice.

Fig. S8

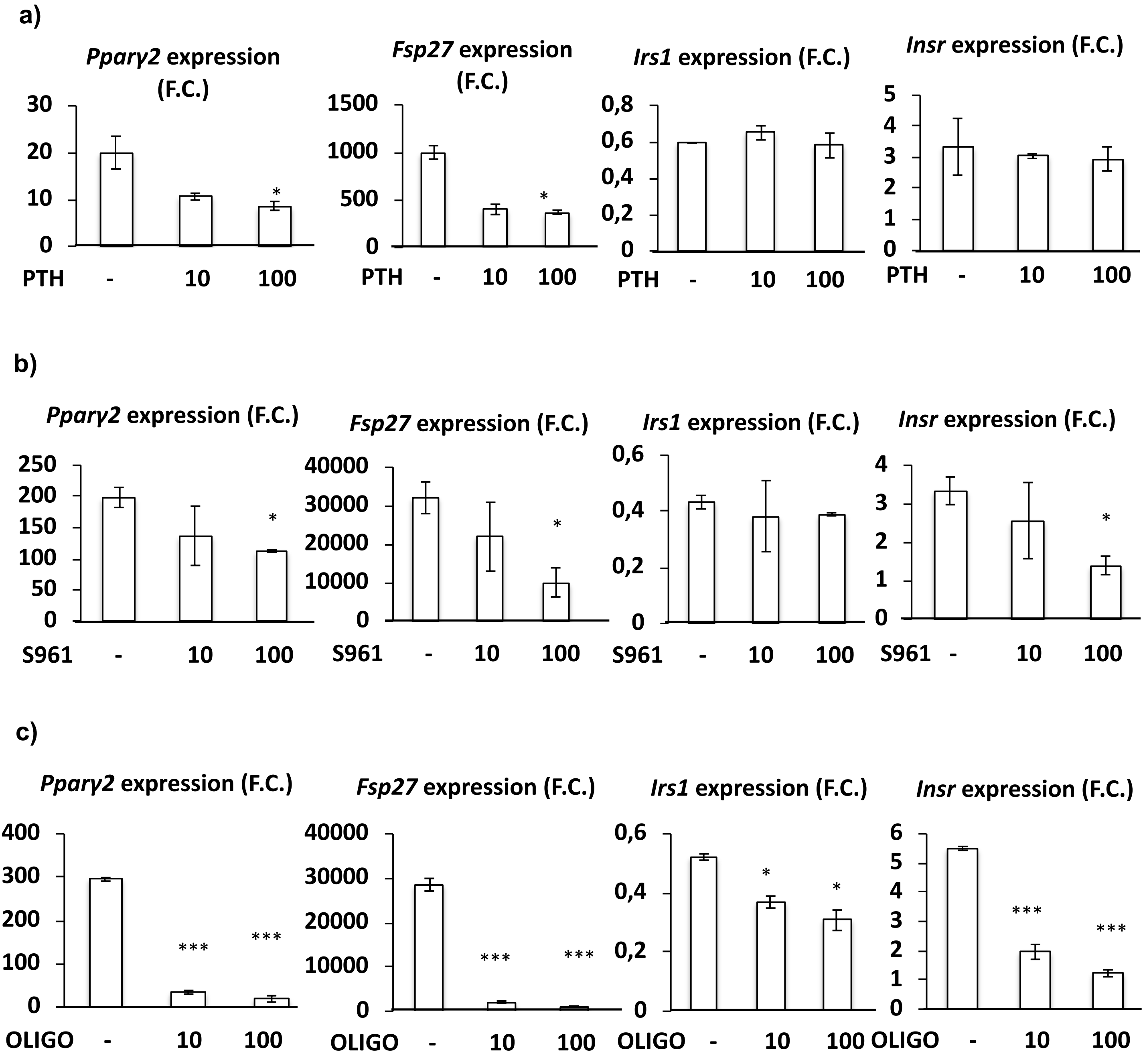

Fig. S8: Effect of PTH, S961 and Oligomycin treatment on adipogenic potential of mBMSC (D10) (a-c). (**a**) Gene expression of *Pparγ2, Fsp27, Irs1 and Insr* after 10d of AD differentiation with PTH treatment; (n=3); (**b**) Gene expression of *Pparγ2, Fsp27, Irs1 and Insr* after 10d of AD differentiation with S961 treatment; (n=3); (**c**) Gene expression of *Pparγ2, Fsp27, Irs1 and Insr* after 10d of AD differentiation with Oligomycin treatment; (n=3). Data are presented as mean fold change (F.C.) of gene expression over undifferentiated cells ± SEM, (n=3 per group); (*p<0.05, ***p< 0.001: AD vs treated cells, two-tailed unpaired Student's t test).

SUPPLEMENTARY TABLE

Table S1. List of primer sequences used for qRT-PCR.

Gene	Forward sequence	Reverse sequence
**36b4**	TCCAGGCTTTGGGCATCA	CTTTATCAGCTGCACATCACTCAGA
**Hprt**	TCAGTCAACGGGGGACATAAA	GGGGCTGTACTGCTTAATCAG
**Il1β**	GCAACTGTTCCTGAACTCAACT	ATCTTTTGGGGTCCGTCAACT
**Tnfα**	CCCTCACACTCAGATCATCTTCT	GCTACGACGTGGGCTACAG
**RelA**	ACTGCCGGGATGGCTACTAT	TCTGGATTCGCTGGCTAATGG
**Lcn2**	GGGAAATATGCACAGGTATCCTC	CATGGCGAACTGGTTGTAGTC
**Irs1**	TCTACACCCGAGACGAACACT	TGGGCCTTTGCCCGATTATG
**Irs2**	CTGCGTCCTCTCCCAAAGTG	GGGGTCATGGGCATGTAGC
**Insr**	ATGGGCTTCGGGAGAGGAT	CTTCGGGTCTGGTCTTGAACA
**Foxo1**	ATGCTCAATCCAGAGGGAGG	ACTCGCAGGCCACTTAGAAAA
**Igf1r**	GTGGGGGCTCGTGTTTCTC	GATCACCGTGCAGTTTTCCA
**Glut1**	GCAGTTCGGCTATAACACTGG	GCGGTGGTTCCATGTTTGATTG
**Glut2**	TTCCAGTTCGGCTATGACATCG	CTGGTGTGACTGTAAGTGGGG
**Glut3**	ACCAAAAGCAACGGAGAAGAG	GGCATTCCGAAACAGGTAACTC
**Glut4**	CTCATGGGCCTAGCCAATGC	CCCTGATGTTAGCCCTGAGTA
**Adipoq**	GACGTTACTACAACTGAAGAGC	CATTCTTTTCCTGATACTGGTC
**Lep**	GAGACCCCTGTGTCGGTTC	CTGCGTGTGTGAAATGTCATTG
**LepR**	GAATGAGCAAGGTCAAAACTGC	CCTAGCTGGCGAAAAACTGAAG
**Fsp27**	ATCAGAACAGCGCAAGAAGA	CAGCTTGTACAGGTCGAAGG
**Cidea**	ATCACAACTGGCCTGGTTACG	TACTACCCGGTGTCCATTTCT
**Hsl**	TGCTTGGTTCAACTGGAGAG	GTAACTGGGATGGCTGCCAT
**Cd36**	ATGGGCTGTGATCGGAACTG	TTTGCCACGTCATCTGGGTTT
**C/ebpα**	AAGCCAAGAAGTCGGTGG A	CAGTTCACGGCTCAGCTGTTC
**Pparγ2**	GGGTCAGCTCTTGTGAATGG	CTGATGCACTGCCTATGAGC
**Alpl**	GCCCTCTCCAAGACATATA	CCATGATCACGTCGATATCC
**Runx2**	AGCAACAGCAACAACAGCAG	GTAATCTGACTCTGTCCTTG
**Opn**	GAAACTCTTCCAAGCAATTC	GGACTAGCTTGTCCTTGTGG
**Oc**	CAGACAAGTCCCACACAGCA	CTTTATTTTGGAGCTGCTGT
**Bmp2**	GGGACCCGCTGTCTTCTAGT	TCAACTCAAATTCGCTGAGGAC
**Pthr1**	CAACTACAGCGAGTGCCTCA	GAGACATGGAATATCCCACGGT
**Ucp1**	CACTCAGGATTGGCCTCTACG	GGGGTTTGATCCCATGCAGA
**Ucp2**	ATGGTTGGTTTCAAGGCCACA	TTGGCGGTATCCAGAGGGAA

**Ucp3**	CTGCACCGCCAGATGAGTTT	ATCATGGCTTGAAATCGGACC
**Prdm16**	CAGCACGGTGAAGCCATTC	GCGTGCATCCGCTTGTG
**Pparα**	AGAGCCCCATCTGTCCTCTC	ACTGGTAGTCTGCAAAACCAAA
**Mttp**	CTCTTGGCAGTGCTTTTTCTCT	GAGCTTGTATAGCCGCTCATT
**p21**	CCTGGTGATGTCCGACCTG	CCATGAGCGCATCGCAATC
**p53**	TCTTATCCGGGTGGAAGGAAA	GGCGAAAAGTCTGCCTGTCTT
**Fasl**	CCGCTCTGATCTCTGGAGTGA	CACGAAGTACAACCCAGTTTCG
**Serpine1**	GGACACCCTCAGCATGTTCA	CGGAGAGGTGCACATCTTTCT
**Serpineb2**	TTCCGCATACTGGAAACATCAG	GGATGCGTCCTCAATCTCATC
**Hmox1**	AGGTACACATCCAAGCCGAGA	CATCACCAGCTTAAAGCCTTCT
**Sod2**	CAGACCTGCCTTACGACTATGG	CTCGGTGGCGTTGAGATTGTT
**Atg7**	TCCGTTGAAGTCCTCTGCTT	CCACTGAGGTTCACCATCCT
**Lc3b**	ACAAAGAGTGGAAGATGTCCGGCT	TGCAAGCGCCGTCTGATTATCTTG
**Lamp1**	TAGTGCCCACATTCAGCATCTCCA	TTCCACAGACCCAAACCTGTCACT
**Beclin1**	GGCCAATAAGATGGGTCTGA	GCTGCACACAGTCCAGAAAA
